# The immunological landscape of peripheral blood in glioblastoma patients and immunological consequences of age and dexamethasone treatment

**DOI:** 10.3389/fimmu.2024.1343484

**Published:** 2024-01-18

**Authors:** Sophie A. Dusoswa, Jan Verhoeff, Saskia van Asten, Joyce Lübbers, Marlous van den Braber, Sophie Peters, Sanne Abeln, Matheus H.W. Crommentuijn, Pieter Wesseling, William Peter Vandertop, Jos W. R. Twisk, Thomas Würdinger, David Noske, Yvette van Kooyk, Juan J. Garcia-Vallejo

**Affiliations:** ^1^ Department of Molecular Cell Biology and Immunology, Amsterdam Infection and Immunity Institute, Cancer Center Amsterdam, Amsterdam UMC, VU Amsterdam, Amsterdam, Netherlands; ^2^ Department of Neurosurgery, Amsterdam UMC, VU Amsterdam, Amsterdam, Netherlands; ^3^ Department of Computer Science, Free University, Amsterdam, Netherlands; ^4^ Department of Pathology, Cancer Center Amsterdam, Brain Tumor Center Amsterdam, Amsterdam and Princes Máxima Center for Pediatric Oncology, Amsterdam UMC, VU Amsterdam, Utrecht, Netherlands; ^5^ Department of Epidemiology and Biostatistics and Biostatistics, Amsterdam Public Health Research Institute, Amsterdam UMC, VU Amsterdam, Amsterdam, Netherlands

**Keywords:** glioblastoma, mass cytometry, immune monitoring, dexamethasone, large cohort

## Abstract

**Background:**

Glioblastomas manipulate the immune system both locally and systemically, yet, glioblastoma-associated changes in peripheral blood immune composition are poorly studied. Age and dexamethasone administration in glioblastoma patients have been hypothesized to limit the effectiveness of immunotherapy, but their effects remain unclear. We compared peripheral blood immune composition in patients with different types of brain tumor to determine the influence of age, dexamethasone treatment, and tumor volume.

**Methods:**

High-dimensional mass cytometry was used to characterise peripheral blood mononuclear cells of 169 patients with glioblastoma, lower grade astrocytoma, metastases and meningioma. We used blood from medically-refractory epilepsy patients and healthy controls as control groups. Immune phenotyping was performed using FlowSOM and t-SNE analysis in R followed by supervised annotation of the resulting clusters. We conducted multiple linear regression analysis between intracranial pathology and cell type abundance, corrected for clinical variables. We tested correlations between cell type abundance and survival with Cox-regression analyses.

**Results:**

Glioblastoma patients had significantly fewer naive CD4+ T cells, but higher percentages of mature NK cells than controls. Decreases of naive CD8+ T cells and alternative monocytes and an increase of memory B cells in glioblastoma patients were influenced by age and dexamethasone treatment, and only memory B cells by tumor volume. Progression free survival was associated with percentages of CD4+ regulatory T cells and double negative T cells.

**Conclusion:**

High-dimensional mass cytometry of peripheral blood in patients with different types of intracranial tumor provides insight into the relation between intracranial pathology and peripheral immune status. Wide immunosuppression associated with age and pre-operative dexamethasone treatment provide further evidence for their deleterious effects on treatment with immunotherapy.

## 1 Introduction

Glioblastoma is the most common primary brain tumor and has a dismal prognosis under the current standard of care including maximal safe resection, radiotherapy, and temozolomide (1, 2). In addition, most patients are treated with steroids for relief of symptoms due to peritumoral vasogenic edema (3, 4). However, the immunological consequences of this treatment are currently unknown and a matter of debate in light of ongoing immunotherapy trials (5–8). The glioblastoma microenvironment contains immune cells with potential for anti-glioblastoma immunity (9, 10) and efforts to boost the anti-tumor immune response with immunotherapy are hopeful, but evidence of sustainable clinical responses in glioblastoma is lacking (11). Clinical trials have shown induction of tumor-specific T cells, but expansion of these cells is poorly correlated with tumor elimination and clinical improvement (11). This discrepancy is suggestive for tumor-imposed suppression of the immunotherapy induced T cell response (12).

Glioblastomas dampen the immune system both locally and systemically (12, 13). For the development of effective anti-glioblastoma immunotherapy we need a comprehensive understanding of glioblastoma-specific weaknesses in the immune system and immunomodulatory effects of dexamethasone (14). The systemic immune composition of patients with diffuse gliomas is influenced by tumor-derived soluble factors such as extracellular vesicles, cytokines and growth factors, and is characterized by systemic T cell lymphopenia and aberrations in the monocytic lineage (13, 15). To date, studies have focused on immunological deficiencies of single cell types and did not take into account the immunomodulatory effects of dexamethasone (14). In practice however, the immune system acts as an interrelated network in which cell types work together and respond to each other. Since dexamethasone is known to affect multiple immune cell types, the immune system is compromised at multiple levels after dexamethasone administration (4, 16).

Recently, a multiparameter flow cytometry analysis of the systemic immune status in glioblastoma patient-derived blood revealed an association between myeloid-derived suppressor cells (MDSC) and worse prognosis (17). In line with this work there is a need for tools for blood-based patient selection and monitoring of immunotherapy effects in clinical trials. For a comprehensive characterization of peripheral blood mononuclear cells (PBMCs) we employed mass cytometry by time of flight (CyTOF), which provides a single cell platform for cytometry with up to 45 markers.

We hypothesized that the peripheral immune composition of glioblastoma patients would be distinct in comparison with patients with lower grade astrocytoma WHO II or WHO III, intra-axial metastases, meningioma, medically refractory epilepsy or healthy controls, and would be affected by treatment with the immunosuppressive corticosteroid dexamethasone and tumor volume. Therefore, we studied the peripheral immune composition of 169 patients with intracranial tumors, epilepsy and healthy controls by CyTOF, and examined the effects of intracranial pathology, dexamethasone treatment, tumor volume, sex, and age on immune composition.

## 2 Materials and methods

### 2.1 Study design and data sources

Between April 2015 and November 2018, we prospectively collected blood from all patients admitted to the Amsterdam UMC with a glioblastoma, lower grade astrocytoma, intra-axial metastasis, meningioma, and medically refractory epilepsy after obtaining written informed consent. Approval for taking blood samples for translational research was obtained from the Medical Ethics Review Committee of the Amsterdam UMC. Blood was drawn in 3 EDTA 6ml tubes (Vacutainer, BD) at the operating room between induction of anesthesia and incision and processed within three hours. For data acquisition we selected all patients who underwent resection of a glioblastoma, lower grade astrocytoma, meningioma or epilepsy surgery. In addition, 20 randomly selected patients who underwent resection of an intra-axial metastasis and 22 randomly selected patients who underwent meningioma resection were included. All groups except patients with medically refractory epilepsy and healthy controls contained a subgroup of patients who received dexamethasone prior to surgery.

### 2.2 Data acquisition

High-dimensional peripheral blood phenotypes were analyzed with data acquired on a CyTOF Helios instrument after antibody stainings (**Supplementary Table 1**) of blood samples in barcoded batches of 20 patients per batch as described in detail in **Supplementary Information**. Clinical data collection from medical files was approved by the Medical Ethics Review Committee of the Amsterdam University Medical Center (UMC) and managed in a deidentified format. Diagnoses were confirmed by the clinical caretakers and clinical pathology reports. IDH mutation status was derived from clinical pathology reports. Progression free survival (PFS) was determined according to Response Assessment in Neuro-Oncology (RANO) criteria and clinical parameters.

### 2.3 Data analysis

Signal intensities and sample acquisition rate were reviewed for stability over time. Pre-analysis gating included Gaussian parameters, viability, CD45 expression, and cell size as described in **Supplementary Figure 1**. CD45+ live cells were further analyzed using R Studio. Data was then normalized with the help of a reference sample included in each batch and using the Cytonorm algorithm^17^. Marker distributions of the reference samples before and after normalization are shown in **Supplementary Figures 2**, **3** respectively. To visualize global sample similarities principal component analysis (PCA) was performed on median values of all markers of individual samples. To improve separation, Random Forest (RF) modelling was run to determine the 5 best classifying markers (CD4, CXCR3, CD7, CD3, CD28) in distinguishing the different clinical groups. The median values of the 5 top distinguishing markers were used to calculate principal components. When splitting clinical groups also based on dexamethasone use as well, RF modelling identified PD-1, TIM-3, CD3, CD2 and CD7 as best performing classifiers. Clusters of phenotypically similar cells were identified using the FlowSOM-package (version 1.4.1) in R 3.5.1. Initial SOM-clustering was set to 1000 clusters, using all markers except the checkpoint inhibitors OX40, FasR, TIM-3, PD-1, CTLA-4, ICOS, LAG-3, and 4-1BB. The 1000 SOM-clusters were meta-clustered into 200 clusters using consensus clustering. The remainder of the data not used in clustering was mapped to the nearest SOM-cluster to obtain the relative abundance of all consensus-clusters across individual patients and median intensity values per cluster. For visualization and cluster interpretation we performed a tSNE dimensionality reduction with perplexity was set at 30, Theta at 0.5, and the number of iterations at 1500. To reach biologically recognizable metaclusters for further analysis we determined and merged similar consensus-clusters in a supervised manner, listed in **Supplementary Table 2**. Through hierarchical clustering of median marker intensities (based on Euclidean distance) and inspecting grouping of consensus clusters on tSNE projection we determined 19 metaclusters. Expression patterns of the resulting metaclusters were compared to canonical cell types for identification and validated using a gating approach shown in **Supplementary Figure 4**. Consensus-clusters with poor similarity to tSNE projection and absent to low expression for phenotyping markers were grouped as unidentified cells. Final visualization of results was performed using optSNE dimensionality reduction after removal of doublets.

### 2.4 Tumor volume

Tumor volumes were determined using the smart brush tool in BrainLab (iPlan Net 3.7.0.64) by delineating the contrast enhancing regions of the tumors in T1 images in sagittal, coronal and axial fields of view.

### 2.5 Statistical analysis

Mean frequencies per patient group were compared using the nonparametric Kruskal-Wallis test for group comparison and Mann-Whitney U tests for *post-hoc* analyses. Multiple test correction through Benjamini-Hochberg adjustment (*α* = 0.1) was applied for the selected pairwise group comparisons referred to in section 3.1 (14 tests and 15 tests respectively). In order to study the relative contribution of pathology (patient group) to the composition of PBMCs we performed linear regression analyses. Separate multivariable linear regression analyses were performed for the 19 cell types with patient groups as independent variables adjusted for age and sex in one model, and age, sex, and pre-operative dexamethasone use in a second model. A third model was constructed adjusting for IDH-mutation status of the patient and whether the tumor was recurrent or a first operation. Residuals were checked for normal distribution and in the case of not-normal distribution of the residuals, the independent variables were Log^10^ transformed. For correct interpretation the regression coefficients obtained from the Log^10^ transformed analyses were transformed back. Statistical tests and models were not corrected for the number of different cell types that were identified. Next, 74 glioblastoma patients were investigated for the relation between tumor mass and cell type abundance. Two analyses were performed: 1) Linear regression analyses to identify the relation between tumor mass and cell types. For these analyses, a forward selection procedure with the 19 cell types was performed. In one version, we take into account age, sex, and IDH mutational status. In the other version we also correct for dexamethasone use. Only statistically significant (*p*<0.05 with α = 0.10) factors remained in the final multivariable models. 2) The effect of cell types on PFS was analyzed with Cox regression analysis. PFS was censored if progression was not observed by the end of the study. Regression analyses were performed in SPSS (version 26). A heatmap was constructed displaying the cell type frequencies per patient with meningioma, glioblastoma or metastases. Per cell type the percentages were centered around the mean and standard deviation was scaled to 1. Sample clustering was based on Euclidean distances and Multi Response Permutation Procedure (MRPP) was performed to assess mean similarity within and between groups. Groups were based on dexamethasone use prior to surgery or on underlying pathology.

## 3 Results

**Figure 1:**
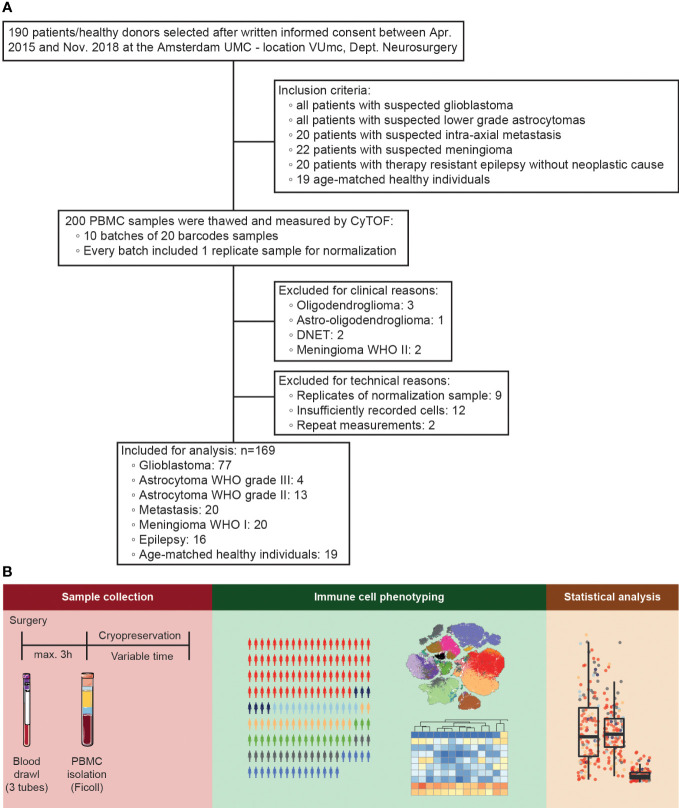
Study design. **(A)** Flow diagram representing inclusion and exclusion of patient samples. 200 peripheral blood mononuclear cell (PBMC) samples (172 patients and 19 healthy donors plus 9 duplicates of the normalization sample) were selected for the study, of which after exclusion for technical, clinical, and data-driven reasons 169 were analyzed. **(B)** Characterization of PBMCs of patients undergoing intracranial surgery by mass cytometry. PBMCs were isolated, cryopreserved, and measured by mass cytometry for population identification and statistical analysis.

In order to analyze the influence of glioblastoma on peripheral immune status we performed a 35-parameter immunophenotyping analysis on PBMCs. As depicted in the flow diagram in **Figure 1A**, blood was collected from 172 patients who underwent resection of a suspected glioblastoma or lower grade astrocytoma, 20 randomly selected patients who underwent resection of an intra-axial metastasis, 22 randomly selected patients who underwent meningioma resection, and all 18 patients who underwent surgery for medically refractory epilepsy. Nineteen healthy donor samples, age-matched to glioblastoma patients, were included as well. Patients were grouped according to pathologically confirmed final diagnoses. Samples were measured in ten batches of 20 samples, including an identical reference sample per batch, which adds up to a total of 200 samples. The reference samples were used to normalize batch differences as previously described^17^. For analysis, nine out of the ten identical normalization samples were excluded, as well as twelve samples with insufficient yields and eight samples with pathologically confirmed final diagnoses that could not be included: oligodendroglioma, astro-oligodendroglioma, DNET, and meningioma WHO II (**Figure 3A**). Lack of sample size for these clinically distinct pathologies would limit generalizability.

Patient demographics and relevant clinical characteristics of the included samples are summarized in **Table 1**. There was a difference in age between the patient groups as determined by one-way ANOVA (p < 10^-6^). Gliobastoma patients were older than patients with epilepsy (p < 10^-6^, Levene’s test p = 0.125) and astrocytoma WHO II (p < 10^-6^). There was no difference between glioblastoma patients and healthy donors (p > 0.99), astrocytoma WHO III (p = 0.221), metastases (p > 0.99), or meningioma WHO I (p > 0.99), and patients with epilepsy versus astrocytoma WHO II (p > 0.99) or astrocytoma WHO III (p > 0.99). The IDH1 R132 mutation was found in 10% of the glioblastomas, 43% of the astrocytomas WHO III, and 77% of the astrocytomas WHO II. Dexamethasone was administered pre-operatively in 74% of the glioblastoma patients, 85% of the patients with an intra-axial metastasis, and 35% of the patients with a meningioma, opposed to none of the patients with a lower grade astrocytoma (WHO II and WHO III) or epilepsy surgery.

**Table 1:** Patient demographics and clinical characteristics.

	Glioblastoma	Astrocytoma WHO III	Astrocytoma WHO II	Metastasis	Meningioma	Epilepsy	Healthy Control	Total
Number of patients	77	4	13	20	20	16	19	169
Male/female #/# (% male))	49/28 (64%)	2/2 (50%)	9/4 (69%)	13/7 (65%)	7/13 (35%)	9/7 (56%)	9/10 (47%)	
Mean age	59	46	31	58	55	36	55	
Newly diagnosed	70	4	10	15	19			
Recurrent (# first/# second)	(5/3)	(0/0)	(2/1)	(5/0)	(1/0)			
IDH mutants (%)	8 (10.4%)	2 (50%)	11 (84.6%)					
Meta lung carcinoma				9				
Meta mamma carcinoma				3				
Meta colorectal cancer				3				
Meta melanoma				2				
Meta other				3				
Dexamethasone use pre-operative (%)	58 (75%)	- (0%)	- (0%)	17 (85%)	7 (35%)	- (0%)	- (0%)	

### 3.1 Peripheral blood immune cell composition is affected by malignancy grade and dexamethasone use

**Figure 2:**
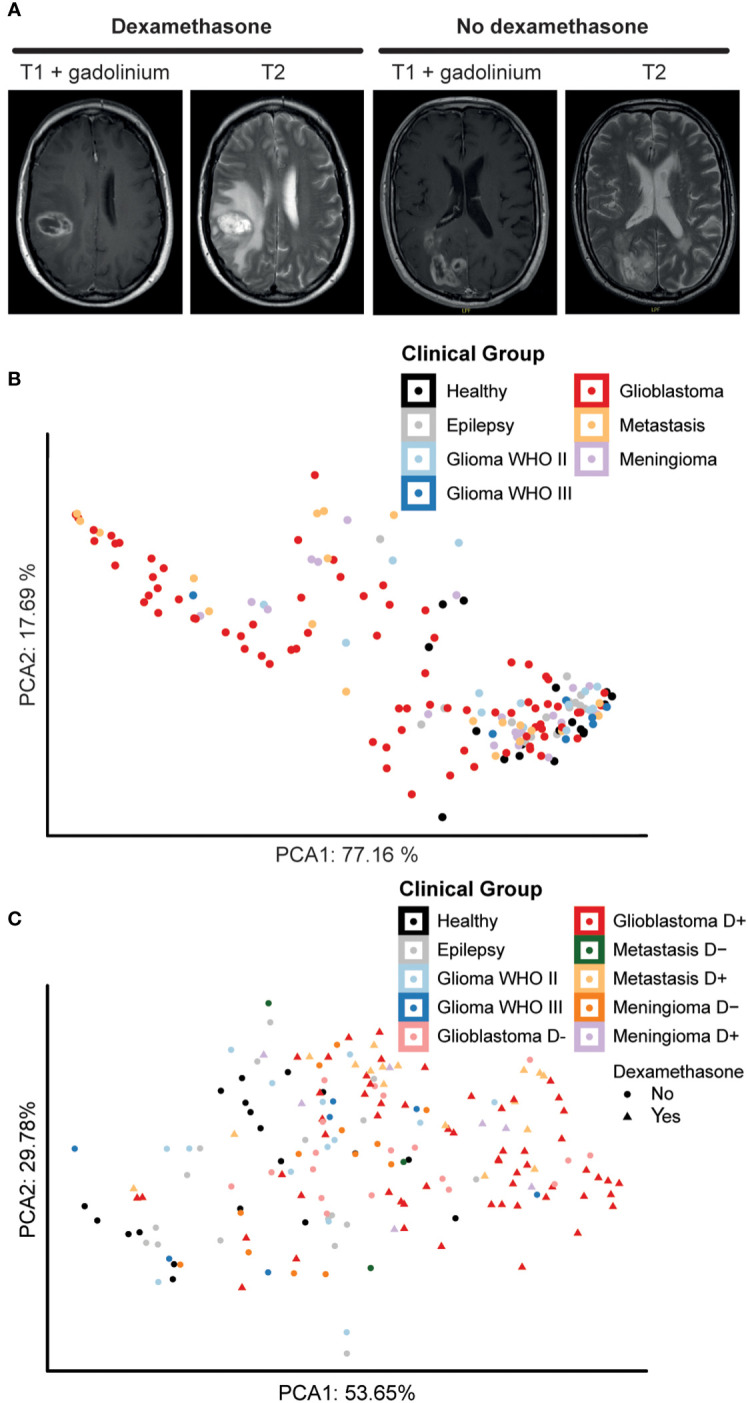
Dexamethasone influences peripheral blood composition of patients with glioblastoma, metastases, and meningioma. **(A)** Axial T1 with gadolinium and T2 MRI pictures showing an example of a patient with extensive peritumoral edema who was treated with dexamethasone pre-operatively and a patient who did not receive dexamethasone prior to surgery because of the relative absence of peritumoral edema. **(B)** Principal component analysis (PCA) of sample-wide median expression levels. Principle components calculated over the 5 markers best distinguishing the different patient groups. Data points representing 169 samples split by intracranial pathology. Axes labels display percentage of variance explained. **(C)** PCA plot of sample-wide median expression levels. Principle components calculated over 5 markers best distinguishing patient groups, here splitting intracranial pathology by dexamethasone treatment before surgery. Data points representing 169 samples colored for intracranial pathology. Patient groups that underwent dexamethasone treatment before surgery are highlighted with a different shape. Axes labels display percentage of variance explained.

Intra-operative blood samples were processed for PBMC isolation, followed by immune cell phenotyping and statistical analysis (**Figure 1B**). **Figure 2A** depicts examples of a patient with a glioblastoma with significant peritumoral edema for which dexamethasone was started before resection and a patient who did not receive dexamethasone. Based on the fact that dexamethasone is a very strong immune suppressor (14), we split the groups with a glioblastoma, intra-axial metastasis, and meningioma into subgroups based on dexamethasone treatment. PBMC characteristics of the patient groups without splitting for dexamethasone treatment depicted in a PCA plot (**Figure 2B**) show a grade of malignancy from right to left with control groups and lower grade malignancies on the right and glioblastoma, meningioma, and metastatis samples to the left. In **Figure 2C** samples were further split based on dexamethasone treatment. Again control groups and lower grade malignancies clustered closely together, now on the left side. Higher malignancy samples without dexamethasone appeared closer to the control groups than the three groups that were treated with dexamethasone, which appeared on the far right side. The pattern observed on the PCA analysis plots indicated that tumor grade and dexamethasone treatment possibly both influenced PBMC composition.

**Figure 3:**
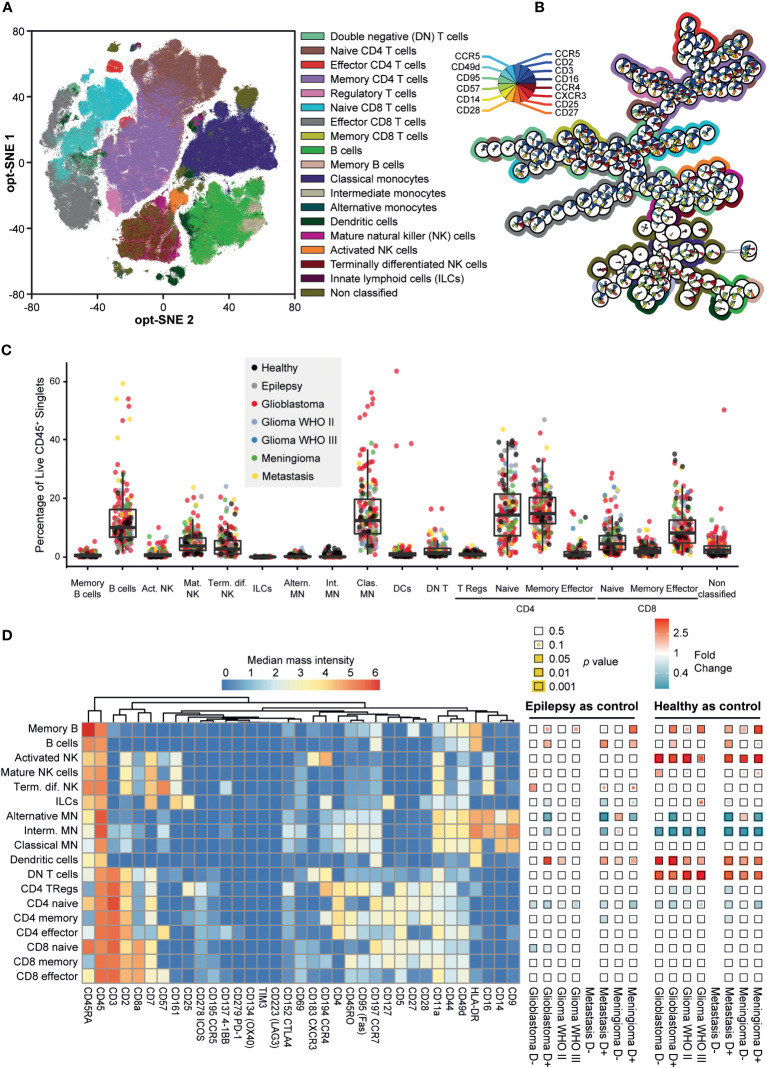
Mass cytometry captures the phenotypic heterogeneity of PBMC samples in patients with intracranial malignancies. **(A)** Opt-SNE mapping of PBMC samples. 10,000 cells were sampled from each patient, with the calculation run on cell classification markers. Data is colored for immune cell types. **(B)** Minimum spanning tree (MST) of 200 FlowSOM metaclusters. Star charts represent a metacluster, filled with median expression levels, normalized to 0-1. Background colors match the immune cell types in **(A)** Metaclusters without background color are CD3^+^/CD14^+^ doublets. **(C)** Boxplot of cell type abundances per sample as a percentage of CD45^+^ live singlets. Datapoints are colored for clinical groups. **(D)** Heatmaps showing the phenotypes of the cell types by expression of 35 markers (left) and fold changes (FC) in abundance of cell types (% of total PBMCs) within each patient group compared to epilepsy and healthy controls (right). Squares are color coded for FC and the statistical significance of two-tailed Mann-Whitney test is visualized as the size of the colored square. P-values are corrected using Benjamini-Hochberg procedure to correct for the 14 pairwise comparisons. FC > 1 (red) indicates cell type abundance is higher in the patient group stated below the heatmap, FC < 1 (blue) indicates cell type is more abundant in the control group stated above.

**Figure 4:**
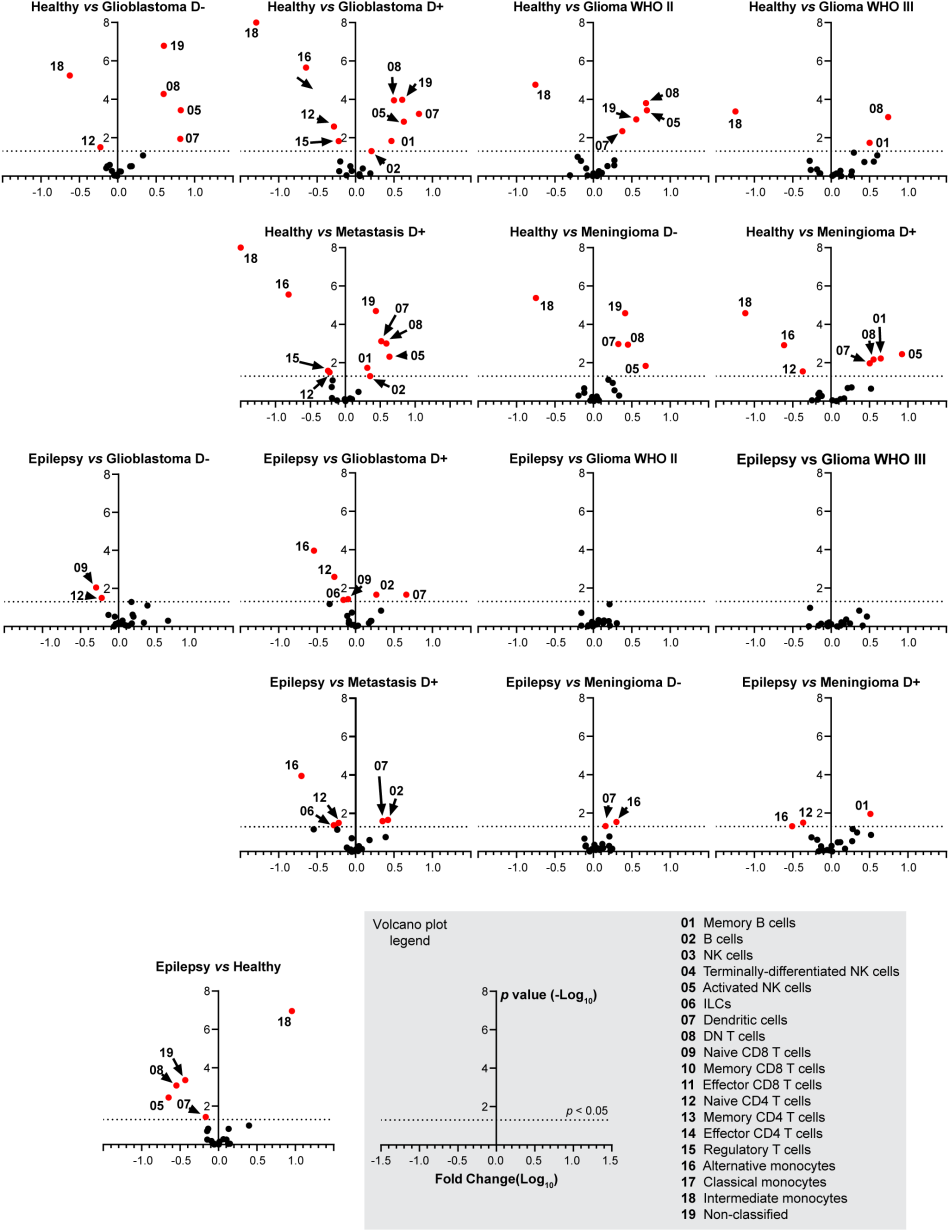
Fold change in cell type abundance comparing healthy and epilepsy controls to patient groups. Data is displayed in volcano plots showing log_10_-transformed FC on the x-axis versus –log_10_(p-value) on the y-axis. Significance level of p < 0.05 is indicated with a dashed line. FC > 1 indicates cell type abundance is higher in the latter named patient group, FC < 1 indicates cell type is more abundant in the control group. D- and D+ represent dexamethasone-naive and dexamethasone treated groups.

Marker expression data of the PBMCs of patient and control samples allowed for clustering and phenotyping, summarized in **Figures 3A, B**. The cohort showed significant heterogeneity within patient groups (**Figure 3C**, **Supplementary Table 4**). **Figure 3D** depicts expression of all the cell surface markers per cell type in a heatmap as an indication of their phenotype. In addition, **Figure 3D** shows the significance of the difference in cell frequency (in percentages) between patients with epilepsy or healthy controls compared to any of the tumor types, color-coded for fold change (see also **Supplementary Table 4** for frequencies and **Figure 4** for fold changes and statistics). Note that the group of patients with metastases without pre-operative dexamethasone treatment includes only three patients, therefore data is excluded.

**Figure 5:**
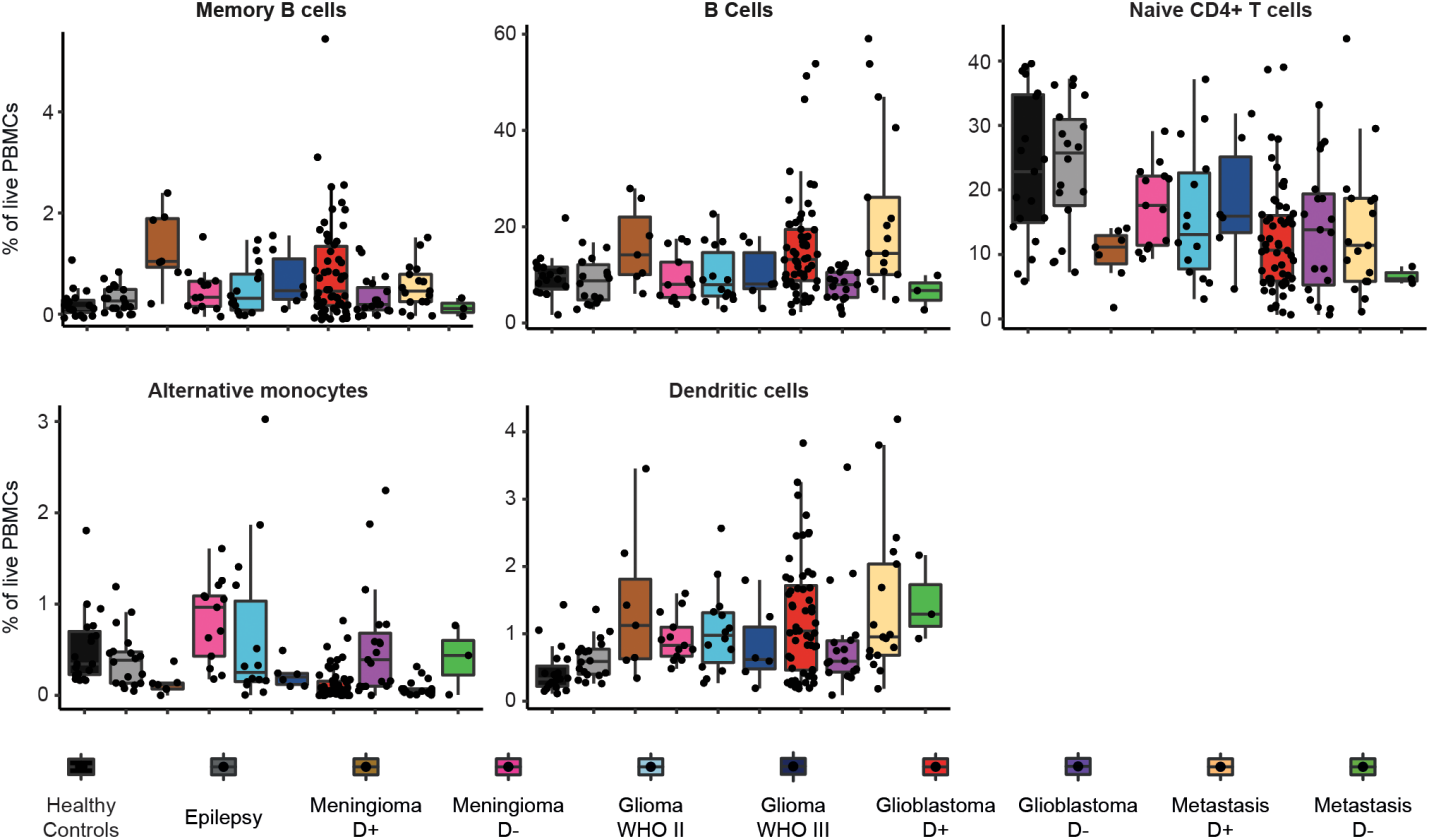
Frequencies of immune cell types in each patient group that showed significant differences between glioblastoma and control groups. Boxplots of percentage per cell type of total live PBMCs split by patient group. Patient groups for glioblastoma, meningioma and metastases are split by dexamethasone-naive (D-) or dexamethasone treated (D+).

Dexamethasone treated patients with glioblastoma, metastases or meningioma have significantly less naive CD4+ T cells and alternative monocytes compared to epilepsy and healthy controls. In addition, dexamethasone treated glioblastoma patients or metastases have significantly more B cells and dendritic cells compared to epilepsy and healthy controls. Dexamethasone treated patients with meningioma have significantly more memory B cells compared to epilepsy and healthy controls. Astrocytoma WHO II and III show a highly similar immune profile compared to epilepsy. **Figure 4** expands on **Figure 3D** by showing volcano plots of the patient groups compared to the control groups. **Figure 5** summarizes the frequencies of the cell types with significant differences per patient group, with glioblastomas, meningiomas and metastases split by dexamethasone treatment. **Supplementary Figure 7** shows the remaining cell types for which fold changes were not statistically significant for both control groups.

### 3.2 Corrected regression analyses identify multiple significant differences in immune subset frequencies between glioblastoma patients and other groups

Multiple regression analyses were performed to establish whether the association between tumor type and immune profile was significantly influenced by dexamethasone use, and to study a possible relation between immune profile and tumor volume. Since patients with a glioblastoma, intra-axial metastasis, or meningioma are generally older compared to patients with a lower grade astrocytoma or epilepsy, multiple regression analyses were corrected not only for sex, but also for age (**Supplementary Table 3**). To study the additional influence of dexamethasone treatment, the multiple regression analyses were repeated corrected for sex, age, and dexamethasone use (**Supplementary Table 3**). In a third iteration, we included IDH mutation status and whether the tumor was a first occurrence or a recurrent episode (data not shown). Regression coefficients, confidence intervals and significance levels are shown in **Supplementary Table 4**. Due to the limited effect of IDH mutation and recurrence these two dependent variables were not included in further analysis.

Our group of interest, glioblastoma patients, was set as constant so the frequencies of the 19 immune cell types can be interpreted relative to glioblastoma. Cell frequencies of four of the cell types were analyzed untransformed; therefore, the regression coefficient could be interpreted as an absolute difference between glioblastoma patients and any of the other diagnoses. Thus, without correcting for any possible confounder, patients with epilepsy had 10.817% more naive CD4+ T cells than our reference group glioblastoma (95% CI: 5.754 – 15.880, p =4.11•10^-5^). For part of the other cell types the cell frequencies were Log^10^ transformed due to non-normal distribution of the residuals, and therefore the regression coefficients were transformed back. In these cases, the regression coefficients must be interpreted as a relative difference, with for example 0.662 times less B cells in patients with epilepsy compared to glioblastoma (95% CI 0.467 – 0.939, p = 0.021) in the uncorrected model.

**Figure 5**, **Supplementary Table 4**, and **Supplementary Figure 7** depict means with standard deviations of cell percentages of all the cell subsets for the different groups.

The analysis of the percentage cells per subsets between groups revealed that glioblastoma patients have fewer more CD4+ T cells than patients with epilepsy (8.483% more in epilepsy, 95% CI: 1.918 – 15.04, p = 0.012), after correction for sex, age, and dexamethasone. Glioblastoma patients also had mature natural killer (NK) cells than patients with epilepsy (0.569 times less in epilepsy patients, 95% CI: 0.337 – 0.962, p = 0.036). Both patterns also hold true for the comparison between glioblastoma and healthy controls, which lie closer in age to glioblastoma patients (8.674% more naive CD4+ T cells in healthy controls, 95% CI: 2.919 – 14.430, p = 0.003 and 0.439 times less mature NK cells in healthy controls, 95% CI: 0.277 – 0.696, p = 0.001). Furthermore, glioblastoma patients have less CD4+ T memory cells (5.537% more in epilepsy, 95% CI: 0.577 – 10.490, p = 0.029) than patients with epilepsy, but this was not the case when compared to healthy controls. In the comparison with healthy controls, glioblastoma patients had more activated NK cells (0.218 times less in healthy controls, 95% CI: 0.103 – 0.461, p = 9.34•10^-5^), more DN T cells, (0.327 times less in healthy controls, 95% CI: 0.198 – 0.541, p = 2.01•10^-5^) more dendritic cells, (0.434 times less in healthy controls, 95% CI: 0.247 – 0.761, p = 0.004) and fewer intermediate monocytes. (10.447 times more in healthy controls, 95% CI: 4.539 – 24.044, p = 1.20•10^-7^)

### 3.3 Dexamethasone use prior to surgery had a distinct effect on immune cell composition

By comparing significant associations in regression models including or excluding confounders, we can investigate the effect of the confounder. For example, we found more memory B cells in glioblastoma patients compared to epilepsy in the model corrected for sex and age (0.488% fewer in epilepsy corrected for sex and age, 95% CI: -0.903 – -0073, p = 0.021) and compared to healthy controls (0.528% fewer in healthy controls corrected for sex and age, 95% CI: -0.876 – -0.179, p = 0.003), but this association was lost when correcting for dexamethasone as well, for both epilepsy (0.057% fewer in epilepsy corrected for sex, age, and dexamethasone, 95% CI: -0.521 – 0.407, p = 0.809) as well as healthy controls (0.106% fewer in healthy controls corrected for sex, age, and dexamethasone, 95% CI: -0.513 – 0.301, p = 0.607). In addition, after correcting for dexamethasone glioblastoma patients did not have fewer alternative monocytes compared to epilepsy patients or healthy controls. This association was significant when correcting for only sex and age (3.341 times more in epilepsy corrected for sex and age, 95% CI: 1.542 – 7.239, p = 0.002, 4.423 times more in healthy controls corrected for sex and age, 95% CI: 2.309 – 8.475, p < 0.001). We observed a similar pattern for B cells in the comparison between glioblastoma patients and epilepsy patients, there was no significant association after correcting for dexamethasone but this association was present when correcting for only sex and age (0.633 times less in epilepsy corrected for sex and age, 95% CI: 0.430 – 0.933, p = 0.021). Abundance of B cells was not different between healthy controls and glioblastoma patients regardless of corrections. Inversely, healthy controls had similar abundance of regulatory T cells compared to glioblastoma patients after correcting for dexamethasone, but only correcting for sex and age showed a decrease in glioblastoma patients (1.875 times more in healthy controls corrected for sex and age, 95% CI: 1.337 – 2.630, p < 0.001). Comparing glioblastoma patients to epilepsy patients showed no significant correlation to T regulatory cells in any of the models.

As stated, these differences were lost when correcting for dexamethasone, suggesting that dexamethasone treatment contributed to a higher percentage of B cells and memory B cells, and lowered percentage of alternative monocytes and regulatory T cells in glioblastoma patients. Glioblastoma patients also had a trend for a lower percentage of innate lymphoid cells (ILCs; 1.660 times more in epilepsy, 95% CI: 0.995 – 2.767, p = 0.051), naive CD8+ T cells (1.995 times more in epilepsy, 95% CI: 1.285 – 3.097, p=0.002), and had an increased percentage of dendritic cells (0.576 times less in epilepsy, 95% CI: 0.352 – 0.942, p = 0.028). These differences were lost when correcting for sex and age, suggesting that age contributes to a loss of ILCs and naive CD8+ T cells and an increase in dendritic cells. This is further strengthened by the fact that these associations were not found when comparing glioblastoma patients to the age-matched healthy controls.

**Figure 6:**
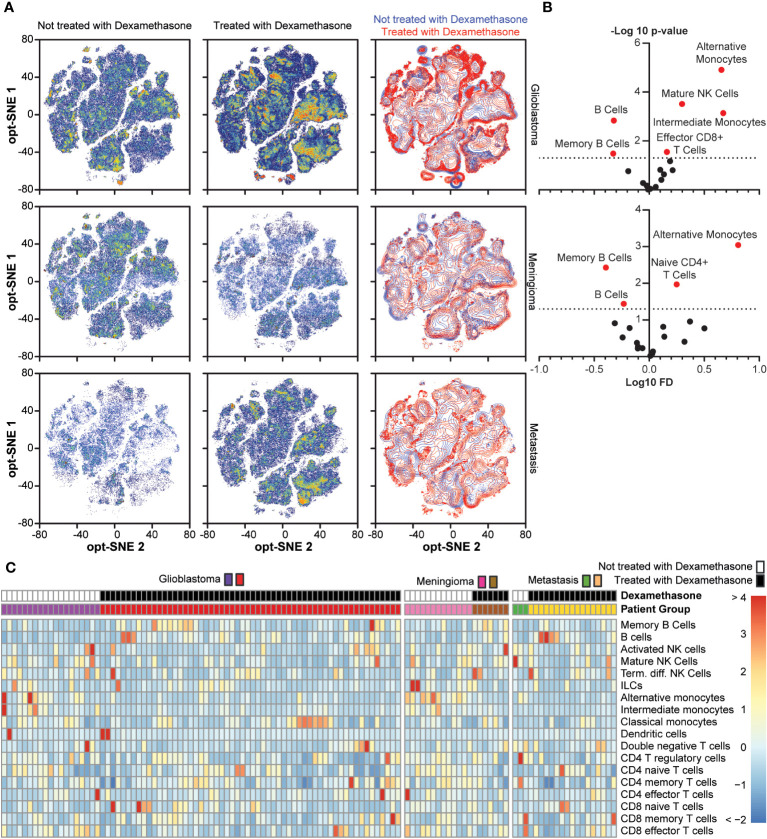
Comparison between dexamethasone treated and not treated in the glioblastoma, meningioma, and metastasis groups. **(A)** Density distribution of cumulative cases per study group depending on dexamethasone treatment in optSNE map, including an overlaid projection. **(B)**. Fold change in cell type abundance comparing dexamethasone treated and untreated in glioblastoma and meningioma patients. Unfortunately, there were not enough cases to make comparisons in the metastasis patient group. Data is displayed in volcano plots showing log_10_-transformed FC on the x-axis versus –log_10_(p-value) on the y-axis. Significance level of p < 0.05 is indicated with a dashed line. FC < 1 indicates cell type is more abundant in the dexamethasone-treated patients. **(C)** Heatmap depicting cell type frequencies per patient in glioblastoma, meningioma, and metastasis patient groups, split based on dexamethasone treatment.

To further investigate the effect of dexamethasone treatment prior to surgery, we directly compared dexamethasone use within the patient groups with glioblastoma, meningioma, and metastases. In **Figure 6A** the density distributions are shown. In both glioblastoma and meningioma groups, memory B cells and B cells had significantly lower abundance in patients not treated with dexamethasone as compared to patients treated with dexamethasone (**Figure 6B**). Cell type frequencies in all patients groups with dexamethasone use are summarized in **Figure 6C**, and a hierarchically clustered version is shown in **Supplementary Figure 8**. The mean distance between patients was 5.716. When we grouped patients by dexamethasone use, regardless of underlying pathology, mean distances were significantly altered (Multi Response Permutation Procedure, p = 0.001). The mean distance within groups dropped to 5.572. Between patients without dexamethasone treatment mean distance was increased to 6.066, between patients with dexamethasone treatment distance dropped to 5.361. Grouping patients based on underlying pathology altered mean distances to a lesser extent: Observed mean distance dropped to 5.684 (p = 0.038), with mean distances within patient groups of 5.732 for glioblastoma, 5.497 for meningioma, and 5.69 for metastasis patients. These results suggested that dexamethasone use prior to surgery has a greater effect on peripheral immune composition than intra-cranial pathology.

### 3.4 Immune composition correlated with tumor characteristics and survival rate

To study the influence of immune composition on PFS in glioblastoma patients we performed a Cox regression analysis on the group of patients with a glioblastoma, stratified on dexamethasone treatment (**Table 2**) and corrected for sex, age, and *IDH1* mutation status. In the group of patients with a glioblastoma without dexamethasone treatment the hazard for progression was higher in patients with higher frequencies of double negative T cells (HR=1.810, 95% CI: 1.016 – 3.198, p = 0.003). In patients treated with dexamethasone the hazard was increased by higher frequencies of regulatory T cells (HR= 2.005, 95% CI: 1.095 – 3.673, p=0.024).

**Table 2:** | Cox regression analysis.

Corrected for: Age, sex, and IDH mutation	Glioblastoma, no dexamethasone				Glioblastoma, dexamethasone treated			
		95% Confidence interval				95% Confidence interval		
	HR	Lower	Upper	p-value	HR	Lower	Upper	p-value
Double Negative T Cells	1.810	1.016	3.198	0.003				
Regulatory T Cells					2.005	1.095	3.673	0.024

Next, we set out to study whether immune composition was also associated with tumor mass and performed a linear regression on the group of patients with a glioblastoma with tumor volume (cm^3^) as dependent variable. With a forward selection procedure for the cell types in this linear regression model, mature NK cells and alternative monocytes were significantly contributing. The frequency of mature NK cells (B = -0.026, 95% CI: -0.048 – -0.005, p = 0.018) and alternative monocytes (B = -0.306, 95% CI: -0.566 – -0.046, p = 0.022) were negatively correlated with tumor mass when we corrected for sex and age. This correlation, however, was not significant anymore when corrected for dexamethasone treatment in the glioblastoma group (**Supplementary Table 5**). Conversely, a negative correlation with memory B cells was revealed after correcting for dexamethasone treatment (B= -1.111, 95% CI: -1.659 – -0.130, p = 0.045).

## 4 Discussion

### 4.1 Presence of intracranial tumors predict peripheral immune composition

This is the largest cohort in which peripheral immune composition of patients with intracranial tumors has been studied on a high dimensional data level. Patient groups were compared to two control groups. Patients with medically refractory epilepsy were a control for the effects of anesthesia, and healthy donor were age-matched to glioblastoma patients. Three conclusions can be drawn from this study. First, in-depth high-dimensional CyTOF analysis of PBMCs in patients with different types of intracranial pathology shows a correlation between malignant intracranial pathology and peripheral immune status. In comparison to healthy donors, glioblastoma patients showed low frequencies of naive CD4+ T cells, CD4+ regulatory T cells, alternative monocytes, and intermediate monocytes, and high frequencies of memory B cells, mature NK cells, activated NK cells, DN T cells, and dendritic cells. Compared to patients with medically refractory epilepsy, patients with a glioblastoma showed low frequencies of naive CD4+ T cells, CD4+ memory T cells, naive CD8+ T cells, and alternative monocytes, and high frequencies of dendritic cells, memory B cells, and B cells. The immune profile of less malignant intracranial tumors such as astrocytoma WHO II and III showed no significant differences. The size of our current cohort allowed for sufficient power to correct for confounders in peripheral immune composition. After these corrections, two significant associations between glioblastoma and subset abundance remained: naive CD4+ T cells and mature NK cells. Glioblastoma is associated with a T cell deficiency in peripheral blood and secondary lymphoid organs (13, 18). We observed a drop in naive CD4+ T cells in glioblastoma compared to both control groups. This is in concert with the findings of Chongsathidkiet et al., who describe exceptionally low T cell numbers, especially of naive CD4+ T cells, due to T cell sequestration in the bone marrow (13). NK cells in glioblastoma are investigated in the context of immunosurveillance (19, 20) or as a vehicle for therapy (21, 22). However little is known about circulating NK cells in intracranial cancer. We observed an increase in peripheral mature NK cells in glioblastoma patients not previously reported. Further characterization of these cells and their potential effect on glioblastoma treatment would need to be investigated.

### 4.2 Immune composition is affected by age, sex, dexamethasone treatment and tumor volume

The second conclusion is that the relation between intracranial pathology and immune composition is strongly influenced by age and pre-operative administration of dexamethasone, and to a lesser extent tumor volume. As stated, we were able to investigate these as confounders in regression analyses. The addition of age, sex, and dexamethasone administration to regression models removed several significant correlations. Though the abundance of CD8+ naive T cells in epilepsy patients was twofold higher compared to glioblastoma patients, after correcting for age in the model no significant association existed between patient group and CD8+ naive T cell abundance. This is in line with the comparison between glioblastoma patients and age-matched healthy controls, here no difference in CD8+ naive T cell abundance was found. Recently, Ladomersky et al. demonstrated an association between human aging and decreased numbers of CD8+ T cells in the blood (23), which could explain the loss of significant difference when correcting for age in the study cohort. Johnson et al. expanded on this, showing a direct link between aging and effective glioblastoma treatment in a mouse model (24), though no data is provided there on peripheral abundance of CD8+ T cells. Therefore, younger patients with a glioblastoma could be more susceptible to T cell directed immunotherapy due to their higher frequency of naive CD8+ T cells at time of surgery.

Addition of dexamethasone administration as a confounder had a greater effect. A significant decrease in alternative monocytes and increase in memory B cells in glioblastoma patients compared to both control groups was lost after correction for dexamethasone use, but not after inclusion of age and sex. Though not found in comparison with healthy controls, glioblastoma patients had a higher mean frequency of B cells compared to epilepsy patients. This association was again lost after correction for dexamethasone use. This suggests that dexamethasone influences frequencies of B memory cells and alternative monocytes and potentially B cells. Tumor volume was smaller in patients with more mature NK cells and alternative monocytes. However, the model corrected for dexamethasone treatment revealed, that tumor volume was smaller in patients with more memory B cells. Again this is evidence for the role in dexamethasone use on alternative monocyte and memory B cell abundance, modulating the association between cell types and tumor volume.

Direct comparisons of glioblastoma and meningioma patients treated with or without dexamethasone showed similar changes, dexamethasone treated patients had a significant decrease in alternative monocytes and significant increases in memory B cells and B cells. Han et al. report an increase of regulatory B cells in glioblastoma (25). Further characterization of B cell subsets and activation status should be investigated in dexamethasone treated patients. Inhibition of regulatory B cells in patients treated with dexamethasone could potentially be beneficial in combinatorial immunotherapy (26). Changes in peripheral monocytes in response to dexamethasone have previously been reported (27). Though we a observed a decrease in alternative monocytes, it is unclear if this can fully account for the reported decreases, since alternative are a smaller subset of all monocytes. However the lowered abundance of these alternative monocytes after dexamethasone treatment is of importance in light of the work by van den Bosssche et al. (28). The authors report a positive association between circulating CD16+ alternative monocytes and overall survival of glioblastoma patients. Since 1961, the synthetic corticosteroid dexamethasone has been the treatment of choice in patients with cerebral edema in the context of a brain tumor (3). Recent studies provide evidence on the negative association between dexamethasone use and overall survival of glioblastoma patients (18, 29–31). While the exact mechanism remains unclear, the strong immunomodulatory effects of dexamethasone that we observed could very well contribute to the poorer overall survival.

To apply efficient immunotherapies in patients with glioblastoma an alternative less immunotoxic option for the treatment of intracranial edema may be needed. Peritumoral edema is caused by blood brain barrier (BBB) destabilization due to the expression of tumor-derived factors such as vascular endothelial growth factor (VEGF) and Angiotensin-II (32, 33). Dexamethasone stabilizes the BBB by regulating Angiopoietin-I and VEGF (34). Alternatives to dexamethasone such as anti-VEGF treatment or Angiotensin-II inhibitors are currently under investigation (35).

### 4.3 Implications for immunotherapy targeting glioblastoma

Aside from the associations between PBMC subsets between intracranial pathology, age, and dexamethasone, the third conclusion is that peripheral immune composition within the groups of patients showed heterogeneity. In part due to these significant confounders, our findings demonstrate that the immune composition in glioblastoma patients is not uniform. Clinical evaluation of peripheral immune status could aid in the selection and evaluation of patients for immunotherapy trials once trial cohorts can be profiled and analyzed to determine immune biomarkers.

Immunological biomarkers have been associated with survival in glioblastoma patients (36, 37). Using clinical lab parameters neutrophils and neutrophil to lymphocyte ratios (NLR) have shown prognostic value for overall survival (37). The clinical lab parameters in these studies, however, lack the resolution to specify the types of lymphocytes. In our cohort, after correcting for sex, age, and *IDH1* mutation status, PFS in dexamethasone-naive glioblastoma patients correlated with double negative T cells, whereas patients treated with dexamethasone showed a correlation between PFS and regulatory T cells. How these cell subsets associated with PFS influence the treatment would need to be investigated but might hold promise.

To better elucidate the relation between dexamethasone treatment and immune composition a prospective trial would need to be set up. Randomizing pre-operative administration of dexamethasone would shed light on whether the associations between dexamethasone use and PFS were causative or were related to the underlying indication for dexamethasone treatment (i.e. peritumoral edema).

Our study design investigated the peripheral immune status of patients with intracranial pathologies. How our findings relate to local tumor microenvironment conditions was not in the scope of this work. Whether the immunosuppression found in the peripheral immune system was a reflection of a cold tumor microenvironment commonly found in glioblastoma (38, 39) could not be verified but would be a promising avenue of research.

## 5 Conclusion

Although some early phase clinical trials for immunotherapy have shown modest effectiveness (7, 40, 41), phase III randomized clinical trials have failed to demonstrate improved overall survival (42–44). This doesn’t mean that there is no perspective for immunotherapy in glioblastoma, especially in younger patients. More comprehensive high-dimensional analyses of the immunobiology in glioblastoma are needed for designing more promising personalized combination immunotherapeutic approaches for this immunologically complex disease. Heterogeneity of immune fingerprints within the group of glioblastoma patients could facilitate a role for high-dimensional CyTOF analysis as tool for monitoring treatment effects and predicting response to treatment (45). In addition, our results on the effect of dexamethasone on the immune composition add to the growing doubts whether it is the correct tool when used in conjunction with immunotherapy. In the context of intracranial tumors, other treatments for peritumoral edema warrant consideration.

## Data availability statement

The original contributions presented in the study are included in the article/**Supplementary Material**, further inquiries can be directed to the corresponding author.

## Ethics statement

The studies involving humans were approved by Medical Ethics Review Committee of the Amsterdam UMC. The studies were conducted in accordance with the local legislation and institutional requirements. The participants provided their written informed consent to participate in this study.

## Author contributions

SD: Conceptualization, Formal analysis, Investigation, Validation, Visualization, Writing – original draft. JV: Formal analysis, Investigation, Methodology, Software, Validation, Visualization, Writing – original draft. SvA: Investigation, Writing – review & editing. JL: Formal analysis, Investigation, Writing – review & editing. MB: Investigation, Writing – review & editing. SP: Software, Validation, Writing – review & editing. SA: Resources, Software, Supervision, Validation, Writing – review & editing. MC: Investigation, Writing – review & editing. PW: Resources, Writing – review & editing. WV: Resources, Writing – review & editing. JT: Formal analysis, Writing – review & editing. TW: Resources, Supervision, Writing – review & editing. DN: Resources, Supervision, Writing – review & editing. YK: Funding acquisition, Supervision, Writing – review & editing. JG-V: Funding acquisition, Project administration, Supervision, Writing – original draft.
