# Supplementary material for: The immunological landscape of peripheral blood in glioblastoma patients and immunological consequences of age and dexamethasone treatment
Source: Front Immunol. 2024 Jan 18;15:1343484. doi: 10.3389/fimmu.2024.1343484 (PMC10839779; doi:10.3389/fimmu.2024.1343484)
Supplement: Supplementary file 1 [file DataSheet_1.docx]

# Supplementary methods: experimental procedures

## Blood samples.

Peripheral blood mononuclear cells (PBMC) were isolated from 3 6mL EDTA tubes (Vacutainer, BD) using a ficoll gradient as described in detail before^1^ and subsequently cryopreserved in liquid nitrogen. Samples were split into 6 aliquots. All samples, including age-matched healthy control volunteers, were collected at the Amsterdam UMC, location VU University Medical Center (Amsterdam, The Netherlands) after approval by The Medical Ethics Review Committee of the Amsterdam UMC and given written informed consent. Patients with epilepsy underwent surgery when resistant to medical treatments; patients with glioblastoma, lower grade astrocytoma, metastases and meningioma underwent surgery as primary treatment followed by radiotherapy and/or chemotherapy according to treatment standards. Samples were grouped according to the diagnoses assigned by a pathologist at the Amsterdam UMC, location VU University Medical Center, and handled according to National and institutional medical ethical standards regarding the use of human tissue.

## Antibodies.

The details of the antibody mass cytometry panel (clone, metal tag and provider) are listed in supplemental table 1. The antibodies that were not purchased from Fluidigm were conjugated using the MAXPAR X8 Metal Labeling Kit (Fluidigm), according to manufacturer’s instructions. After conjugation, antibody recovery was determined on a spectrophotometer (Nanodrop, ThermoFisher) at 280nm and validated in a test staining with a positive control.

## Antibody staining and barcoding.

Cryopreserved PBMCs were thawed, washed with thawing medium and subsequently with phosphate-buffered saline (PBS). Samples were manually counted to assess yield. For samples with fewer than 5x10^5^ live cells an additional aliquot was thawed and added. For live cell discrimation after CyTOF measurement we incubated with 5µM Cell-ID cisplatin (Fluidigm) for 5 minutes at room temperature (RT). Cisplatin was quenched with Cell Staining Buffer (CSB, Fluidigm) and washed twice in CSB, then incubated with Human TruStain FcX™ Fc Receptor Blocking Solution (Biolegend) for 10 min at RT. Next, cells were incubated for 30 min with a cocktail of metal-conjugated antibodies against cell surface markers (CCR4, CCR5, CXCR3, CD127) that are sensitive to fixation (Supplementary figure 9). Following 2 washing steps with CSB, cells were fixed with 1.6% paraformaldehyde (PFA) for 15 min. Further antibody staining steps were according to standard experimental protocols for mass tag barcoding, surface antibody staining, and intracellular staining provided by Fluidigm. In short, cells were permeabilized by washing with Maxpar Barcode Perm Buffer (Fluidigm) and incubated with mass tag barcodes in permeabilization buffer for 45 min. Then, cells were incubated with antibodies against surface targets for 30 min at room temperature After washing with CSB, antibodies were fixed with 1.6% PFA for 15 min. For staining of intracellular target CTLA-4 cells were permeabilised by washing twice with Perm-S buffer (Fluidigm). Cells were incubated with antibodies for intracellular markers for 30 min at room temperature in Perm-S buffer. The different antibody cocktails are outlined in Supplementary table 1. After two washing steps with CSB cells were incubated overnight with ^191/193^Ir DNA intercalator (1:4000) diluted in Fix-and-Perm Buffer (Fluidigm) at 4°C. The next day cells were washed twice with CSB and 2x with H_2_O before acquisition on the CyTOF3-Helios.

## Mass cytometry data acquisition.

After washing in H_2_O, samples were diluted in 0.1X EQ Four Element Calibration Beads and were acquired on a CyTOF Helios mass cytometer at a rate of 200-250 events/second. Data was normalized using bead normalization^2^. Deconvolution of pooled samples was performed by processing FCS files with the standard single-cell debarcoding algorithm for CyTOF data^3^.

List of software and packages (versions) used in quality control and cytometry data analysis

Cytobank^4^

R (3.5.1)^5^

Rstudio (1.1.456)^6^

FlowSOM (1.4.1)^7^

FlowCore (1.52.1)^8^

pheatmap (1.0.12)^9^

caret (6.0-85)^10^

dpylr (0.8.5)^11^

ggplot2 (3.3.0)^12^

reshape2 (1.4.3)^13^

Rtsne (0.15)^14^

RColorBrewer (1.1-2)^15^

vegan (2.5-6)^16^

# References

1 Horrevorts SK, Stolk DA, van de Ven R, *et al.* Glycan-modified melanoma-derived apoptotic extracellular vesicles as antigen source for anti-tumor vaccination. *Cancers (Basel)* 2019; **11**. DOI:10.3390/cancers11091266.

2 Finck R, Simonds EF, Jager A, *et al.* Normalization of mass cytometry data with bead standards. *Cytom Part A* 2013; **83 A**: 483–94.

3 Zunder ER, Finck R, Behbehani GK, *et al.* Palladium-based mass tag cell barcoding with a doublet-filtering scheme and single-cell deconvolution algorithm. *Nat Protoc* 2015; **10**: 316–33.

4 Kotecha N, Krutzik PO, Irish JM. Web-Based Analysis and Publication of Flow Cytometry Experiments. *Curr Protoc Cytom* 2010; **53**: 10.17.1-10.17.24.

5 Team RDC. A Language and Environment for Statistical Computing. *R Found Stat Comput* 2018; **2**: https://www.R-project.org.

6 RStudio T. RStudio: Integrated Development for R. RStudio, PBC, Boston, MA URL http://www.rstudio.com/. 2020.

Gretton A, Sch B. A Kernel Method for the Two-Sample-Problem. *Adv Neural Inf Process Syst* 2006; **19**: 513–20.

7 Van Gassen S, Callebaut B, Van Helden MJ, *et al.* FlowSOM: Using self-organizing maps for visualization and interpretation of cytometry data. *Cytom Part A* 2015; **87**: 636–45.

Gretton A, Borgwardt K, Rasch M, Schölkopf B, Smola A. A Kernel Two-Sample Test Arthur. *J Mach Learn Res* 2012; **13**: 723–73.

8 Meur N Le, Hahne F, Ellis B. FlowCore: data structures package for flow cytometry data. 2007.

9 Kolde R. Pheatmap: Pretty Heatmaps (version 1.0. 12). 2019.

10 Kuhn M. Package ‘caret’. The R journal. R J. 2020.

11 Wickham H. dplyr: A grammar of data manipulation. R package version 0.4. 2015.

12 Wickham H. ggplot2: elegant graphics for data analysis. *Springer* 2016.

13 Wickham H. Journal of Statistical Software Reshaping Data with the reshape Package. 2007 http://www.jstatsoft.org/.

14 Krijthe JH. Rtsne: T-distributed stochastic neighbor embedding using Barnes-Hut implementation. R package version 0.13. 2015.

15 Neuwirth E, Brewer RC. ColorBrewer palettes. R package version p. 1.1-2. 2014.

16 Warton, D. I., Wright, S. T., & Wang, Y. (2012). Distance‐based multivariate analyses confound location and dispersion effects. *Methods in Ecology and Evolution*, 3(1), 89-101.

# Supplementary tables

## Supplementary table 1.

Onco-immunology T cell focussed antibody panel

| Target protein | Clone | Metal | Source | Staining step | Cell type | Function |
| --- | --- | --- | --- | --- | --- | --- |
| Cell identification |  |  |  |  |  |  |
| Barcodes |  | 103-110Pd | Fluidigm |  | Staining standardization & doublet discrimination |  |
| Iridium |  | 191-193Ir | Fluidigm |  | Cell identification |  |
| Cisplatin |  | 194-195Pt | Fluidigm |  | Live/dead discrimination |  |
| Cell classification |  |  |  |  |  |  |
| CD45 | HI30 | 89Y | Fluidigm | Multiplexed surface staining | All immune cells | Regulator of T- and B cell receptor signaling |
| CD49d | 9F10 | 141Pr | Fluidigm | Multiplexed surface staining | T cells, B cells, dendritic cells, macrophages, NK cells | Integrin for homing |
| CD11a | HI111 | 142Nd | Fluidigm | Multiplexed surface staining | B cells (dim), monocytes (high) |  |
| CD5 | UCHT2 | 143Nd | Fluidigm | Multiplexed surface staining | T cells and regulatory B cells | Negative regulator of T cell receptor |
| CD195 (CCR5) | NP-6G4 | 144Nd | Fluidigm | *Before fixation* | T cells, macrophages, granulocytes | Transendothelial migration during inflammation |
| CD4 | RPA-T4 | 145Nd | Fluidigm | Multiplexed surface staining | CD4^+^ T helper cells |  |
| CD8a | RPA-T8 | 146Nd | Fluidigm | Multiplexed surface staining | CD8^+^ cytotoxic T cells |  |
| CD7 | CD7-6B7 | 147Sm | Fluidigm | Multiplexed surface staining | T cells, NK cells | Aids in T cell interactions |
| CD25 (IL-2R) | 2A3 | 149Sm | Fluidigm | Multiplexed surface staining | T cells, B cells, NK cells, macrophages | IL-2 receptor |
| CD2 | TS1/8 | 151Eu | Fluidigm | Multiplexed surface staining | T cells, B cells NK cells | Cell adhesion to other cells |
| CD14 | 61D3 | 154Sm | eBioscience | Multiplexed surface staining | Monocytes, macrophages, granulocytes | LPS receptor |
| CD183 (CXCR3) | G025H7 | 156Gd | Fluidigm | *Before fixation* | T cells (CD8+ Tcm), NK cells | Chemokine receptor, chemotaxis, adhesion |
| CD194 (CCR4) | L291H4 | 158Gd | Fluidigm | *Before fixation* | T cells (Th2 cells), B cells, dendritic cells, macrophages | Migration in response to SDF-1 |
| CD197 (CCR7) | G043H7 | 159Tb | Fluidigm | Multiplexed surface staining | T cells (cm and naive), B cells |  |
| CD28 | CD28.2 | 160Gd | Fluidigm | Multiplexed surface staining | T cells | Costimulatory molecule on T cells |
| CD69 | FN50 | 162Dy | Fluidigm | Multiplexed surface staining | T cells | Actvation marker |
| CD161 | HP3G10 | 164Dy | Fluidigm | Multiplexed surface staining | NK cells | Inhibits NK cell cytotoxicity. Enhances T-cell proliferation |
| CD45RO | UCHL1 | 165Ho | Fluidigm | Multiplexed surface staining | T cells (memory) |  |
| CD44 | BJ18 | 166Er | Fluidigm | Multiplexed surface staining | B cells (dim), monocytes, mDCs | Cell adhesion and migration |
| CD27 | 323 | 167Er | Fluidigm | Multiplexed surface staining | T cells | Costimulatory molecule on T cells |
| CD45RA | HI100 | 169Tm | Fluidigm | Multiplexed surface staining | B cells, T cells (effector and naive) |  |
| CD3 | UCHT1 | 170Er | Fluidigm | Multiplexed surface staining | T cells | T cell receptor |
| CD9 | SN4 C3-3A2 | 171Yb | Fluidigm | Multiplexed surface staining | Monocytes, basophils, activated T & B cells | Platelet aggregation |
| CD57 | HCD57 | 172Yb | Fluidigm | Multiplexed surface staining | CD8^+^ T cells |  |
| HLA-DR | L243 | 174Yb | Fluidigm | Multiplexed surface staining | B cells, mDCs, | Antigen presentation |
| CD127 (IL-7Ra) | A019D5 | 176Yb | Fluidigm | *Before fixation* | T cell stimulation | IL/7 receptor, important for T cell development |
| CD16 | 3G8 | 209Bi | Fluidigm | Multiplexed surface staining | T cells, dendritic cells, NK cells | Fc receptor, mediates phagocytosis |
| Immuno-Oncology |  |  |  |  |  |  |
| CD134 (OX40) | ACT35 | 150Nd | Fluidigm | Multiplexed surface staining | T cells | Suppresses apoptosis |
| CD95 (Fas) | DX2 | 152Sm | Fluidigm | Multiplexed surface staining | T cells, B cells, Monocytes, Macrophages, NK cells | Apoptosis induction via binding to FAS ligand |
| CD366 (TIM-3) | F382E2 | 153Eu | Fluidigm | Multiplexed surface staining | T cells, dendritic cells, monocytes, macrophages | Inhibits Th1 mediated immune responses |
| CD279 (PD-1) | EH12.2H7 | 155Gd | Fluidigm | Multiplexed surface staining | T cells, B cells, monocytes, macrophages | Expressed on pro-B cells and apoptosis induction in T cells |
| CD152 (CTLA-4) | 14D3 | 161Dy | Fluidigm | *After permeabilization* | T cells, B cells | T cell inhibition |
| CD278 (ICOS) | C398.4A | 168Er | Fluidigm | Multiplexed surface staining | T cells | Costimulatory molecule important for Th2 cells |
| CD137 (4-1BB) | 4B4-1 | 173Yb | Fluidigm | Multiplexed surface staining | T cells, NK cells | Costimulatory molecule |
| CD223 (LAG-3) | 11C3C65 | 175Lu | Fluidigm | Multiplexed surface staining | T cells, NK cells | Costimulatory molecule |

## Supplementary table 2.

FlowSOM cluster phenotypes overlaid on tSNE

| FlowSOM | Phenotype | FlowSOM | Phenotype | FlowSOM | Phenotype |
| --- | --- | --- | --- | --- | --- |
| 1 | CD4 naive T cells | 71 | CD8 memory T cells | 141 | Non-classical monocytes |
| 2 | CD4 naive T cells | 72 | Classical monocytes | 142 | CD8 effector T cells |
| 3 | CD4 naive T cells | 73 | CD8 memory T cells | 143 | CD8 effector T cells |
| 4 | CD4 naive T cells | 74 | CD8 memory T cells | 144 | CD8 effector T cells |
| 5 | CD4 memory T cells | 75 | CD8 effector T cells | 145 | CD8 effector T cells |
| 6 | CD4 memory T cells | 76 | Double negative T cells | 146 | Double negative T cells |
| 7 | CD4 memory T cells | 77 | Double negative T cells | 147 | Non-classified |
| 8 | CD4 memory T cells | 78 | CD4 T regulatory cells | 148 | Non-classical monocytes |
| 9 | CD4 naive T cells | 79 | CD4 memory T cells | 149 | CD8 effector T cells |
| 10 | CD4 memory T cells | 80 | Double negative T cells | 150 | Double negative T cells |
| 11 | CD4 memory T cells | 81 | Non-classified | 151 | Double negative T cells |
| 12 | CD4 memory T cells | 82 | Classical monocytes | 152 | Activated NK cells |
| 13 | CD4 memory T cells | 83 | Intermediate monocytes | 153 | Activated NK cells |
| 14 | CD4 memory T cells | 84 | CD8 memory T cells | 154 | Non-classified |
| 15 | CD4 memory T cells | 85 | CD8 effector T cells | 155 | Classical monocytes |
| 16 | CD4 memory T cells | 86 | CD4 memory T cells | 156 | Classical monocytes |
| 17 | CD4 memory T cells | 87 | Non-classified | 157 | Dendritic cells |
| 18 | CD4 memory T cells | 88 | Non-classified | 158 | Terminally differentiated NK Cells |
| 19 | CD4 memory T cells | 89 | CD8 effector T cells | 159 | Double negative T cells |
| 20 | CD8 naive T cells | 90 | Double negative T cells | 160 | CD8 effector T cells |
| 21 | CD4 memory T cells | 91 | CD8 effector T cells | 161 | Mature NK Cells |
| 22 | CD4 memory T cells | 92 | Double negative T cells | 162 | Terminally differentiated NK Cells |
| 23 | CD4 effector T cells | 93 | Non-classified | 163 | Activated NK cells |
| 24 | CD4 T regulatory cells | 94 | Classical monocytes | 164 | B cells |
| 25 | CD4 naive T cells | 95 | CD8 effector T cells | 165 | Non-classified |
| 26 | CD4 naive T cells | 96 | CD8 effector T cells | 166 | B cells |
| 27 | CD4 T regulatory cells | 97 | Double negative T cells | 167 | Debris-Doublets |
| 28 | CD4 T regulatory cells | 98 | CD8 effector T cells | 168 | Classical monocytes |
| 29 | CD4 memory T cells | 99 | CD8 effector T cells | 169 | Classical monocytes |
| 30 | CD4 memory T cells | 100 | Classical monocytes | 170 | Dendritic cells |
| 31 | CD4 memory T cells | 101 | Classical monocytes | 171 | Debris-Doublets |
| 32 | CD4 memory T cells | 102 | CD8 effector T cells | 172 | Terminally differentiated NK Cells |
| 33 | CD8 naive T cells | 103 | CD8 memory T cells | 173 | Terminally differentiated NK Cells |
| 34 | CD8 naive T cells | 104 | CD8 memory T cells | 174 | Double negative T cells |
| 35 | Double negative T cells | 105 | CD8 memory T cells | 175 | Mature NK Cells |
| 36 | CD4 naive T cells | 106 | CD8 effector T cells | 176 | Mature NK Cells |
| 37 | CD4 memory T cells | 107 | CD8 effector T cells | 177 | Terminally differentiated NK Cells |
| 38 | CD4 memory T cells | 108 | CD8 effector T cells | 178 | Non-classified |
| 39 | CD8 naive T cells | 109 | CD8 effector T cells | 179 | B cells |
| 40 | CD8 naive T cells | 110 | CD8 effector T cells | 180 | B cells |
| 41 | CD8 naive T cells | 111 | CD8 effector T cells | 181 | Terminally differentiated NK Cells |
| 42 | CD4 T regulatory cells | 112 | CD8 effector T cells | 182 | Non-classified |
| 43 | CD8 naive T cells | 113 | CD8 effector T cells | 183 | Mature NK Cells |
| 44 | CD4 memory T cells | 114 | Double negative T cells | 184 | Mature NK Cells |
| 45 | CD4 memory T cells | 115 | Classical monocytes | 185 | Mature NK Cells |
| 46 | CD4 T regulatory cells | 116 | Classical monocytes | 186 | Classical monocytes |
| 47 | CD4 naive T cells | 117 | CD8 memory T cells | 187 | Terminally differentiated NK Cells |
| 48 | CD4 effector T cells | 118 | CD8 effector T cells | 188 | Mature NK Cells |
| 49 | CD4 effector T cells | 119 | CD8 effector T cells | 189 | Terminally differentiated NK Cells |
| 50 | CD8 naive T cells | 120 | CD8 effector T cells | 190 | Mature NK Cells |
| 51 | CD4 memory T cells | 121 | Non-classified | 191 | Mature NK Cells |
| 52 | CD4 effector T cells | 122 | Classical monocytes | 192 | Memory B Cells |
| 53 | CD4 effector T cells | 123 | CD8 effector T cells | 193 | Double negative T cells |
| 54 | CD4 effector T cells | 124 | CD8 effector T cells | 194 | Mature NK Cells |
| 55 | CD4 memory T cells | 125 | Non-classified | 195 | Non-classified |
| 56 | CD4 naive T cells | 126 | Dendritic cells | 196 | Non-classified |
| 57 | CD4 effector T cells | 127 | Classical monocytes | 197 | Mature NK Cells |
| 58 | CD4 effector T cells | 128 | CD8 effector T cells | 198 | ILCs |
| 59 | CD8 naive T cells | 129 | CD8 effector T cells | 199 | Dendritic cells |
| 60 | Non-classified | 130 | CD8 effector T cells | 200 | B cells |
| 61 | Debris-Doublets | 131 | CD8 effector T cells |  |  |
| 62 | Classical monocytes | 132 | Non-classified |  |  |
| 63 | Intermediate monocytes | 133 | Double negative T cells |  |  |
| 64 | Debris-Doublets | 134 | Non-classified |  |  |
| 65 | Debris-Doublets | 135 | Classical monocytes |  |  |
| 66 | CD8 naive T cells | 136 | Intermediate monocytes |  |  |
| 67 | CD8 naive T cells | 137 | CD8 effector T cells |  |  |
| 68 | Classical monocytes | 138 | CD8 effector T cells |  |  |
| 69 | Classical monocytes | 139 | Double negative T cells |  |  |
| 70 | CD8 memory T cells | 140 | Non-classified |  |  |

## Supplementary table 3.

Multiple linear regression analyses. Bolded values highlighted in red denote significant p-values > 0.05, bolded values with white background denote p-value > 0.1 .

| **In glioblastoma**  **% of PBMCs** | | **Uncorrected** | | | | **Corrected for age and sex** | | | | **Corrected for age, sex, and Dex.** | | | |
| --- | --- | --- | --- | --- | --- | --- | --- | --- | --- | --- | --- | --- | --- |
|  |  | 95% Confidence interval | | | | 95% Confidence interval | | | | 95% Confidence interval | | | |
| *All shown as (%), untransformed* | | **B** | **Lower** | **Upper** | **p-value** | **B** | **Lower** | **Upper** | **p-value** | **B** | **Lower** | **Upper** | **p-value** |
| **B memory** | Epilepsy | -0.373 | -0.744 | -0.002 | **0.049** | -0.488 | -0.903 | -0.073 | **0.021** | -0.057 | -0.521 | 0.407 | 0.809 |
| **0.8 %, SD 0.86** | Meningioma | 0.050 | -0.289 | 0.388 | 0.773 | -0.011 | -0.356 | 0.334 | 0.950 | 0.223 | -0.134 | 0.579 | 0.219 |
|  | Gr. II Glioma | -0.142 | -0.547 | 0.263 | 0.489 | -0.254 | -0.711 | 0.204 | 0.275 | 0.176 | -0.324 | 0.675 | 0.488 |
|  | Gr. III Glioma | 0.192 | -0.500 | 0.884 | 0.585 | 0.096 | -0.609 | 0.800 | 0.789 | 0.525 | -0.193 | 1.243 | 0.151 |
|  | Metastasis | -0.120 | -0.491 | 0.251 | 0.524 | -0.113 | -0.483 | 0.257 | 0.548 | -0.250 | -0.614 | 0.114 | 0.177 |
|  | Healthy | -0.487 | -0.833 | -0.141 | **0.006** | -0.528 | -0.876 | -0.179 | **0.003** | -0.106 | -0.513 | 0.301 | 0.607 |
| **Naive CD4 T cells** | Epilepsy | 10.817 | 5.754 | 15.88 | **0.000** | 10.40 | 4.746 | 16.06 | **0.000** | 8.483 | 1.918 | 15.04 | **0.012** |
| **12.30 %, SD 8.50** | Meningioma | 2.710 | -1.915 | 7.335 | 0.249 | 1.906 | -2.805 | 6.616 | 0.425 | 0.864 | -4.178 | 5.906 | 0.735 |
|  | Gr. II Glioma | 5.826 | 0.300 | 11.35 | **0.039** | 5.729 | -0.506 | 11.964 | **0.071** | 3.817 | -3.243 | 10.87 | 0.287 |
|  | Gr. III Glioma | -0.013 | -9.463 | 9.438 | 0.998 | -0.534 | -10.142 | 9.075 | 0.913 | -2.446 | -12.605 | 7.712 | 0.635 |
|  | Metastasis | 2.239 | -2.824 | 7.302 | 0.384 | 2.375 | -2.673 | 7.422 | 0.354 | 2.985 | -2.168 | 8.138 | 0.254 |
|  | Healthy | 11.021 | 6.301 | 15.74 | **0.000** | 10.551 | 5.804 | 15.299 | **0.000** | 8.674 | 2.919 | 14.43 | **0.003** |
| **Memory CD4 T cells** | Epilepsy | 4.539 | 0.747 | 8.331 | **0.019** | 5.822 | 1.564 | 10.080 | **0.008** | 5.537 | 0.577 | 10.49 | **0.029** |
| **15.32 %, SD 7.05** | Meningioma | 0.697 | -2.768 | 4.161 | 0.692 | 0.991 | -2.554 | 4.535 | 0.582 | 0.837 | -2.973 | 4.646 | 0.665 |
|  | Gr. II Glioma | 0.596 | -3.543 | 4.735 | 0.777 | 2.036 | -2.656 | 6.727 | 0.393 | 1.753 | -3.581 | 7.086 | 0.517 |
|  | Gr. III Glioma | 2.690 | -4.388 | 9.769 | 0.454 | 3.646 | -3.585 | 10.876 | 0.321 | 3.363 | -4.312 | 11.03 | 0.388 |
|  | Metastasis | -4.528 | -8.320 | -0.735 | **0.020** | -4.535 | -8.332 | -0.737 | **0.020** | -4.444 | -8.337 | -0.551 | **0.026** |
|  | Healthy | 2.050 | -1.486 | 5.586 | 0.254 | 2.293 | -1.279 | 5.865 | 0.207 | 2.015 | -2.333 | 6.364 | 0.361 |
| **Effector CD4 T cells** | Epilepsy | -0.153 | -1.471 | 1.165 | 0.819 | 0.759 | -0.694 | 2.213 | 0.304 | 0.472 | -1.219 | 2.162 | 0.582 |
| **1.47 %, SD 2.52** | Meningioma | 0.324 | -0.880 | 1.528 | 0.596 | 0.493 | -0.717 | 1.703 | 0.422 | 0.337 | -0.962 | 1.635 | 0.609 |
|  | Gr. II Glioma | -0.413 | -1.851 | 1.026 | 0.572 | 0.632 | -0.970 | 2.233 | 0.437 | 0.345 | -1.473 | 2.163 | 0.708 |
|  | Gr. III Glioma | 1.920 | -0.540 | 4.380 | 0.125 | 2.587 | 0.119 | 5.055 | **0.040** | 2.301 | -0.316 | 4.917 | **0.084** |
|  | Metastasis | -0.011 | -1.329 | 1.307 | 0.987 | -0.008 | -1.304 | 1.288 | 0.990 | 0.083 | -1.244 | 1.410 | 0.901 |
|  | Healthy | 0.387 | -0.842 | 1.616 | 0.535 | 0.538 | -0.682 | 1.757 | 0.385 | 0.257 | -1.226 | 1.739 | 0.733 |

| **In glioblastoma**  **% of PBMCs** | | **Uncorrected** | | | | **Corrected for age and sex** | | | | **Corrected for age, sex, and Dex.** | | | |
| --- | --- | --- | --- | --- | --- | --- | --- | --- | --- | --- | --- | --- | --- |
|  |  | 95% Confidence interval | | | | 95% Confidence interval | | | | 95% Confidence interval | | | |
| *All shown as (%, Log10 transformed)* | | **10^B** | **Lower** | **Upper** | **p-value** | **10^B** | **Lower** | **Upper** | **p-value** | **10^B** | **Lower** | **Upper** | **p-value** |
| **B cells** | Epilepsy | 0.662 | 0.467 | 0.939 | **0.021** | 0.633 | 0.430 | 0.933 | **0.021** | 1.026 | 0.671 | 1.570 | 0.905 |
| **13.68 %, SD 10.03** | Meningioma | 0.915 | 0.665 | 1.260 | 0.585 | 0.847 | 0.614 | 1.169 | 0.311 | 1.100 | 0.794 | 1.525 | 0.564 |
|  | Gr. II Glioma | 0.808 | 0.552 | 1.184 | 0.272 | 0.797 | 0.520 | 1.220 | 0.294 | 1.287 | 0.815 | 2.033 | 0.277 |
|  | Gr. III Glioma | 0.845 | 0.440 | 1.623 | 0.611 | 0.801 | 0.415 | 1.546 | 0.506 | 1.295 | 0.670 | 2.500 | 0.439 |
|  | Metastasis | 1.627 | 1.147 | 2.308 | **0.007** | 1.648 | 1.167 | 2.328 | **0.005** | 1.414 | 1.013 | 1.974 | **0.042** |
|  | Healthy | 0.806 | 0.582 | 1.117 | 0.194 | 0.770 | 0.557 | 1.066 | 0.115 | 1.234 | 0.850 | 1.791 | 0.267 |
| **NK cells** | Epilepsy | 0.912 | 0.604 | 1.375 | 0.657 | 0.836 | 0.526 | 1.327 | 0.445 | 0.569 | 0.336 | 0.962 | **0.036** |
| **5.24 %, SD 4.50** | Meningioma | 1.225 | 0.842 | 1.783 | 0.286 | 1.196 | 0.813 | 1.757 | 0.361 | 0.971 | 0.648 | 1.453 | 0.884 |
|  | Gr. II Glioma | 1.096 | 0.700 | 1.716 | 0.686 | 0.997 | 0.599 | 1.660 | 0.990 | 0.680 | 0.386 | 1.196 | 0.179 |
|  | Gr. III Glioma | 1.090 | 0.507 | 2.347 | 0.824 | 1.021 | 0.465 | 2.239 | 0.959 | 0.696 | 0.309 | 1.569 | 0.380 |
|  | Metastasis | 0.954 | 0.633 | 1.439 | 0.821 | 0.955 | 0.632 | 1.443 | 0.827 | 1.080 | 0.715 | 1.631 | 0.715 |
|  | Healthy | 0.651 | 0.444 | 0.955 | **0.028** | 0.639 | 0.433 | 0.942 | **0.024** | 0.439 | 0.277 | 0.696 | **0.001** |
| **Term. diff. NK** | Epilepsy | 0.587 | 0.328 | 1.052 | 0.073 | 0.661 | 0.343 | 1.274 | 0.214 | 0.545 | 0.254 | 1.167 | 0.117 |
| **3.90 %, SD 3.88** | Meningioma | 1.130 | 0.664 | 1.923 | 0.652 | 1.191 | 0.690 | 2.056 | 0.528 | 1.072 | 0.597 | 1.923 | 0.815 |
|  | Gr. II Glioma | 1.014 | 0.537 | 1.919 | 0.964 | 1.143 | 0.555 | 2.355 | 0.714 | 0.942 | 0.416 | 2.138 | 0.887 |
|  | Gr. III Glioma | 0.938 | 0.316 | 2.786 | 0.908 | 1.033 | 0.339 | 3.148 | 0.955 | 0.851 | 0.262 | 2.767 | 0.787 |
|  | Metastasis | 1.318 | 0.736 | 2.360 | 0.350 | 1.312 | 0.731 | 2.355 | 0.361 | 1.396 | 0.767 | 2.535 | 0.273 |
|  | Healthy | 1.462 | 0.849 | 2.518 | 0.169 | 1.517 | 0.875 | 2.630 | 0.137 | 1.256 | 0.644 | 2.449 | 0.503 |
| **Act. NK cells** | Epilepsy | 1.000 | 0.521 | 1.923 | 0.997 | 1.038 | 0.498 | 2.168 | 0.920 | 0.838 | 0.356 | 1.972 | 0.684 |
| **1.12 %, SD 1.63** | Meningioma | 1.315 | 0.724 | 2.388 | 0.365 | 1.282 | 0.695 | 2.366 | 0.423 | 1.143 | 0.593 | 2.203 | 0.690 |
|  | Gr. II Glioma | 1.265 | 0.619 | 2.576 | 0.518 | 1.340 | 0.596 | 3.013 | 0.478 | 1.081 | 0.432 | 2.716 | 0.865 |
|  | Gr. III Glioma | 1.225 | 0.362 | 4.150 | 0.742 | 1.247 | 0.357 | 4.355 | 0.728 | 1.007 | 0.268 | 3.784 | 0.992 |
|  | Metastasis | 0.955 | 0.498 | 1.837 | 0.890 | 0.962 | 0.499 | 1.854 | 0.906 | 1.028 | 0.526 | 2.014 | 0.933 |
|  | Healthy | 0.272 | 0.148 | 0.500 | **0.000** | 0.269 | 0.145 | 0.499 | **0.000** | 0.218 | 0.103 | 0.461 | **0.000** |
| **ILCs** | Epilepsy | 1.660 | 0.995 | 2.767 | **0.052** | 1.140 | 0.650 | 1.995 | 0.648 | 0.933 | 0.483 | 1.799 | 0.834 |
| **0.05 %, SD 0.05** | Meningioma | 1.432 | 0.891 | 2.307 | 0.137 | 1.337 | 0.832 | 2.153 | 0.229 | 1.191 | 0.713 | 1.995 | 0.501 |
|  | Gr. II Glioma | 1.439 | 0.824 | 2.512 | 0.200 | 0.923 | 0.497 | 1.714 | 0.797 | 0.755 | 0.372 | 1.531 | 0.434 |
|  | Gr. III Glioma | 3.784 | 1.459 | 9.817 | **0.006** | 2.897 | 1.119 | 7.499 | **0.029** | 2.377 | 0.867 | 6.516 | **0.092** |
|  | Metastasis | 0.462 | 0.278 | 0.771 | **0.003** | 0.461 | 0.280 | 0.760 | **0.003** | 0.491 | 0.294 | 0.818 | **0.007** |
|  | Healthy | 1.271 | 0.782 | 2.065 | 0.333 | 1.194 | 0.738 | 1.928 | 0.469 | 0.984 | 0.551 | 1.762 | 0.959 |
| **DN T cells** | Epilepsy | 1.191 | 0.769 | 1.845 | 0.429 | 1.151 | 0.703 | 1.884 | 0.573 | 0.991 | 0.560 | 1.758 | 0.977 |
| **2.40 %, SD 2.81** | Meningioma | 0.979 | 0.658 | 1.459 | 0.919 | 0.959 | 0.637 | 1.445 | 0.841 | 0.885 | 0.570 | 1.374 | 0.582 |
|  | Gr. II Glioma | 1.365 | 0.847 | 2.198 | 0.200 | 1.321 | 0.767 | 2.275 | 0.312 | 1.140 | 0.615 | 2.109 | 0.677 |
|  | Gr. III Glioma | 2.198 | 0.971 | 4.966 | **0.059** | 2.133 | 0.923 | 4.920 | **0.076** | 1.837 | 0.759 | 4.457 | 0.177 |
|  | Metastasis | 1.064 | 0.687 | 1.648 | 0.780 | 1.067 | 0.687 | 1.656 | 0.772 | 1.119 | 0.714 | 1.754 | 0.623 |
|  | Healthy | 0.384 | 0.255 | 0.577 | **0.000** | 0.378 | 0.251 | 0.573 | **0.000** | 0.327 | 0.198 | 0.541 | **0.000** |
| **Naive CD8s** | Epilepsy | 1.995 | 1.285 | 3.097 | **0.002** | 1.009 | 0.656 | 1.552 | 0.965 | 1.086 | 0.658 | 1.795 | 0.746 |
| **5.31 %, SD 5.75** | Meningioma | 1.327 | 0.887 | 1.982 | 0.167 | 1.084 | 0.757 | 1.549 | 0.662 | 1.127 | 0.766 | 1.656 | 0.542 |
|  | Gr. II Glioma | 1.837 | 1.138 | 2.965 | **0.013** | 0.875 | 0.545 | 1.409 | 0.582 | 0.942 | 0.550 | 1.614 | 0.826 |
|  | Gr. III Glioma | 2.014 | 0.887 | 4.571 | **0.094** | 1.194 | 0.575 | 2.483 | 0.631 | 1.285 | 0.592 | 2.793 | 0.524 |
|  | Metastasis | 1.538 | 0.991 | 2.382 | **0.055** | 1.556 | 1.059 | 2.286 | **0.024** | 1.521 | 1.026 | 2.254 | **0.037** |
|  | Healthy | 1.178 | 0.782 | 1.774 | 0.429 | 1.009 | 0.703 | 1.449 | 0.958 | 1.084 | 0.698 | 1.683 | 0.717 |
| **Mem CD8 T cells** | Epilepsy | 1.045 | 0.701 | 1.556 | 0.827 | 1.245 | 0.802 | 1.936 | 0.326 | 1.282 | 0.767 | 2.143 | 0.339 |
| **2.48 %, SD 1.89** | Meningioma | 1.079 | 0.750 | 1.552 | 0.681 | 1.180 | 0.818 | 1.702 | 0.372 | 1.199 | 0.809 | 1.778 | 0.363 |
|  | Gr. II Glioma | 1.117 | 0.724 | 1.722 | 0.616 | 1.324 | 0.817 | 2.153 | 0.253 | 1.365 | 0.787 | 2.371 | 0.267 |
|  | Gr. III Glioma | 1.510 | 0.719 | 3.170 | 0.275 | 1.746 | 0.826 | 3.690 | 0.143 | 1.799 | 0.813 | 3.981 | 0.146 |
|  | Metastasis | 0.762 | 0.513 | 1.135 | 0.180 | 0.755 | 0.509 | 1.119 | 0.159 | 0.748 | 0.500 | 1.119 | 0.156 |
|  | Healthy | 1.005 | 0.693 | 1.455 | 0.978 | 1.069 | 0.738 | 1.545 | 0.724 | 1.099 | 0.701 | 1.726 | 0.677 |
| **Effector CD8** | Epilepsy | 1.169 | 0.809 | 1.690 | 0.403 | 1.291 | 0.853 | 1.950 | 0.224 | 0.962 | 0.600 | 1.542 | 0.869 |
| **8.89 %, SD 6.24** | Meningioma | 1.256 | 0.895 | 1.758 | 0.185 | 1.315 | 0.931 | 1.854 | 0.119 | 1.119 | 0.780 | 1.611 | 0.538 |
|  | Gr. II Glioma | 1.472 | 0.984 | 2.198 | **0.059** | 1.626 | 1.030 | 2.564 | **0.037** | 1.213 | 0.729 | 2.014 | 0.456 |
|  | Gr. III Glioma | 1.679 | 0.845 | 3.342 | 0.138 | 1.820 | 0.902 | 3.673 | **0.094** | 1.358 | 0.653 | 2.818 | 0.410 |
|  | Metastasis | 0.818 | 0.566 | 1.183 | 0.286 | 0.815 | 0.564 | 1.178 | 0.275 | 0.895 | 0.618 | 1.297 | 0.556 |
|  | Healthy | 1.368 | 0.971 | 1.928 | 0.073 | 1.413 | 1.000 | 2.000 | **0.051** | 1.059 | 0.700 | 1.603 | 0.783 |
| **Tregs** | Epilepsy | 1.172 | 0.813 | 1.687 | 0.393 | 1.462 | 0.977 | 2.188 | **0.065** | 1.146 | 0.721 | 1.824 | 0.561 |
| **1.47 %, SD 0.57** | Meningioma | 1.274 | 0.912 | 1.774 | 0.156 | 1.374 | 0.982 | 1.923 | **0.064** | 1.205 | 0.843 | 1.722 | 0.303 |
|  | Gr. II Glioma | 0.989 | 0.664 | 1.472 | 0.953 | 1.250 | 0.802 | 1.950 | 0.324 | 0.982 | 0.596 | 1.618 | 0.942 |
|  | Gr. III Glioma | 1.222 | 0.618 | 2.415 | 0.561 | 1.452 | 0.733 | 2.884 | 0.283 | 1.143 | 0.557 | 2.339 | 0.716 |
|  | Metastasis | 0.893 | 0.619 | 1.285 | 0.540 | 0.887 | 0.619 | 1.271 | 0.512 | 0.957 | 0.665 | 1.380 | 0.818 |
|  | Healthy | 1.770 | 1.259 | 2.489 | **0.001** | 1.875 | 1.337 | 2.630 | **0.000** | 1.479 | 0.984 | 2.218 | **0.059** |
| **Alt. monocytes** | Epilepsy | 2.794 | 1.387 | 5.632 | **0.004** | 3.341 | 1.542 | 7.239 | **0.002** | 1.038 | 0.459 | 2.348 | 0.928 |
| **0.22 %, SD 0.38** | Meningioma | 3.828 | 1.989 | 7.367 | **0.000** | 4.536 | 2.352 | 8.748 | **0.000** | 2.332 | 1.227 | 4.434 | **0.010** |
|  | Gr. II Glioma | 2.578 | 1.202 | 5.530 | **0.015** | 2.934 | 1.254 | 6.865 | **0.013** | 0.915 | 0.381 | 2.198 | 0.841 |
|  | Gr. III Glioma | 2.529 | 0.692 | 9.245 | 0.159 | 3.014 | 0.820 | 11.076 | **0.096** | 0.942 | 0.269 | 3.301 | 0.925 |
|  | Metastasis | 0.431 | 0.214 | 0.869 | **0.019** | 0.421 | 0.211 | 0.839 | **0.014** | 0.638 | 0.335 | 1.216 | 0.171 |
|  | Healthy | 3.955 | 2.055 | 7.611 | **0.000** | 4.423 | 2.309 | 8.475 | **0.000** | 1.411 | 0.689 | 2.890 | 0.344 |
| **Int. monocytes** | Epilepsy | 1.368 | 0.640 | 2.917 | 0.418 | 1.567 | 0.675 | 3.631 | 0.295 | 0.698 | 0.270 | 1.803 | 0.455 |
| **0.16 %, SD 0.36** | Meningioma | 2.198 | 1.099 | 4.406 | **0.026** | 2.541 | 1.259 | 5.140 | **0.010** | 1.652 | 0.798 | 3.428 | 0.175 |
|  | Gr. II Glioma | 0.684 | 0.299 | 1.567 | 0.367 | 0.752 | 0.297 | 1.897 | 0.542 | 0.336 | 0.121 | 0.931 | **0.036** |
|  | Gr. III Glioma | 1.349 | 0.330 | 5.521 | 0.674 | 1.545 | 0.372 | 6.427 | 0.546 | 0.693 | 0.161 | 2.992 | 0.621 |
|  | Metastasis | 0.485 | 0.227 | 1.035 | **0.061** | 0.471 | 0.222 | 1.002 | **0.050** | 0.618 | 0.292 | 1.306 | 0.206 |
|  | Healthy | 21.038 | 10.351 | 42.658 | **0.000** | 22.961 | 11.298 | 46.559 | **0.000** | 10.447 | 4.539 | 24.044 | **0.000** |
| **Class. monocytes** | Epilepsy | 0.700 | 0.468 | 1.047 | **0.082** | 0.731 | 0.466 | 1.146 | 0.170 | 0.723 | 0.429 | 1.222 | 0.224 |
| **18.72 %, SD 13.45** | Meningioma | 0.813 | 0.562 | 1.175 | 0.267 | 0.869 | 0.597 | 1.265 | 0.460 | 0.865 | 0.578 | 1.291 | 0.475 |
|  | Gr. II Glioma | 0.689 | 0.444 | 1.069 | **0.096** | 0.701 | 0.428 | 1.151 | 0.160 | 0.695 | 0.395 | 1.222 | 0.204 |
|  | Gr. III Glioma | 0.498 | 0.234 | 1.054 | **0.069** | 0.522 | 0.243 | 1.122 | **0.095** | 0.518 | 0.230 | 1.164 | 0.111 |
|  | Metastasis | 0.780 | 0.521 | 1.167 | 0.225 | 0.771 | 0.516 | 1.153 | 0.204 | 0.774 | 0.513 | 1.167 | 0.220 |
|  | Healthy | 0.766 | 0.526 | 1.114 | 0.161 | 0.796 | 0.546 | 1.161 | 0.235 | 0.789 | 0.498 | 1.247 | 0.309 |
| **Dendritic Cells** | Epilepsy | 0.576 | 0.352 | 0.942 | **0.028** | 0.664 | 0.382 | 1.154 | 0.146 | 0.815 | 0.429 | 1.548 | 0.530 |
| **2.93 %, SD 9.19** | Meningioma | 0.939 | 0.599 | 1.472 | 0.783 | 0.955 | 0.603 | 1.513 | 0.843 | 1.067 | 0.652 | 1.746 | 0.794 |
|  | Gr. II Glioma | 0.843 | 0.493 | 1.443 | 0.532 | 0.998 | 0.543 | 1.836 | 0.996 | 1.225 | 0.615 | 2.440 | 0.562 |
|  | Gr. III Glioma | 0.742 | 0.296 | 1.858 | 0.521 | 0.821 | 0.321 | 2.099 | 0.678 | 1.007 | 0.373 | 2.715 | 0.989 |
|  | Metastasis | 1.126 | 0.688 | 1.841 | 0.635 | 1.128 | 0.689 | 1.848 | 0.629 | 1.057 | 0.639 | 1.748 | 0.828 |
|  | Healthy | 0.349 | 0.220 | 0.552 | **0.000** | 0.355 | 0.223 | 0.565 | **0.000** | 0.434 | 0.247 | 0.761 | **0.004** |
| **Undefined cells** | Epilepsy | 0.830 | 0.537 | 1.282 | 0.400 | 0.839 | 0.513 | 1.371 | 0.482 | 0.638 | 0.362 | 1.125 | 0.119 |
| **4.14 %, SD 5.99** | Meningioma | 0.817 | 0.548 | 1.216 | 0.317 | 0.818 | 0.543 | 1.233 | 0.335 | 0.705 | 0.457 | 1.089 | 0.115 |
|  | Gr. II Glioma | 0.993 | 0.617 | 1.596 | 0.976 | 1.005 | 0.585 | 1.726 | 0.986 | 0.766 | 0.416 | 1.406 | 0.386 |
|  | Gr. III Glioma | 0.752 | 0.333 | 1.694 | 0.488 | 0.757 | 0.329 | 1.746 | 0.511 | 0.577 | 0.240 | 1.384 | 0.216 |
|  | Metaasis | 0.899 | 0.582 | 1.390 | 0.631 | 0.899 | 0.581 | 1.393 | 0.633 | 0.982 | 0.630 | 1.531 | 0.933 |
|  | Healthy | 0.336 | 0.223 | 0.504 | **0.000** | 0.336 | 0.222 | 0.507 | **0.000** | 0.257 | 0.156 | 0.423 | **0.000** |

## Supplementary table 4.

Immune cell type frequencies per patient group

|  | **GBM no dexa** | | **Glioblastoma dexa** | | **A3 (no dexa)** | | **A2 (no dexa)** | | **Meta No Dexa** | | **Meta Dexa** | | **Mening. no dexa** | | **Meningioma dexa** | | **Epilepsy** | | **Healthy Control** | |
| --- | --- | --- | --- | --- | --- | --- | --- | --- | --- | --- | --- | --- | --- | --- | --- | --- | --- | --- | --- | --- |
|  | Mean | SD | Mean | SD | Mean | SD | Mean | SD | Mean | SD | Mean | SD | Mean | SD | Mean | SD | Mean | SD | Mean | SD |
| Memory B Cells | 0.43 | 0.37 | 0.92 | 0.95 | 0.70 | 0.55 | 0.62 | 0.47 | 0.25 | 0.17 | 0.65 | 0.43 | 0.56 | 0.40 | 1.39 | 0.72 | 0.41 | 0.25 | 0.31 | 0.26 |
| B Cells | 7.48 | 3.40 | 15.71 | 10.65 | 10.14 | 5.41 | 10.12 | 5.73 | 6.45 | 3.58 | 22.10 | 17.30 | 9.40 | 4.90 | 16.04 | 8.34 | 8.57 | 4.37 | 9.76 | 4.13 |
| NK Cells | 8.36 | 6.10 | 4.21 | 3.30 | 3.74 | 1.56 | 4.80 | 2.54 | 11.73 | 10.72 | 5.21 | 4.41 | 6.38 | 5.24 | 6.24 | 3.00 | 4.49 | 4.57 | 3.39 | 2.46 |
| Terminally differentiated NK Cells | 5.31 | 4.13 | 3.44 | 3.71 | 2.65 | 2.91 | 4.25 | 6.06 | 2.42 | 1.50 | 5.33 | 5.33 | 3.49 | 3.00 | 7.18 | 7.28 | 2.92 | 3.87 | 5.44 | 4.78 |
| Activated NK Cells | 1.58 | 2.38 | 0.97 | 1.29 | 0.69 | 0.54 | 1.03 | 1.02 | 1.94 | 1.07 | 0.97 | 0.99 | 1.09 | 1.08 | 1.90 | 2.34 | 1.01 | 1.55 | 0.23 | 0.23 |
| ILCs | 0.06 | 0.06 | 0.04 | 0.04 | 0.13 | 0.16 | 0.05 | 0.03 | 0.04 | 0.02 | 0.03 | 0.04 | 0.11 | 0.13 | 0.03 | 0.04 | 0.06 | 0.04 | 0.05 | 0.04 |
| DN T Cells | 2.88 | 3.49 | 2.24 | 2.56 | 3.50 | 1.88 | 3.34 | 2.84 | 5.44 | 2.47 | 2.74 | 2.58 | 2.00 | 1.44 | 2.53 | 2.32 | 2.33 | 1.38 | 0.71 | 0.33 |
| Naive CD8 T Cells | 3.75 | 3.17 | 5.83 | 6.31 | 12.10 | 8.16 | 7.86 | 5.52 | 3.25 | 1.38 | 6.46 | 6.12 | 5.75 | 2.51 | 5.39 | 4.44 | 7.96 | 4.01 | 5.19 | 3.58 |
| Memory CD8 T Cells | 2.25 | 1.18 | 2.56 | 2.04 | 2.83 | 1.29 | 2.49 | 1.28 | 5.05 | 3.75 | 2.27 | 2.17 | 2.30 | 1.41 | 2.65 | 1.22 | 2.45 | 1.94 | 2.26 | 1.32 |
| Effector CD8 T Cells | 11.60 | 6.52 | 8.00 | 5.94 | 11.53 | 7.31 | 11.22 | 4.07 | 13.92 | 14.30 | 8.38 | 6.38 | 11.93 | 6.74 | 8.71 | 5.11 | 9.29 | 4.99 | 13.04 | 10.23 |
| Naive CD4 T Cells | 12.50 | 8.92 | 12.23 | 8.44 | 17.44 | 9.42 | 17.97 | 11.14 | 6.63 | 1.34 | 13.86 | 10.65 | 17.71 | 6.40 | 9.98 | 4.33 | 23.63 | 10.11 | 23.32 | 11.55 |
| Memory CD4 T Cells | 14.72 | 4.31 | 15.51 | 7.77 | 17.97 | 6.57 | 16.90 | 6.64 | 23.42 | 12.02 | 11.40 | 5.86 | 17.58 | 6.51 | 13.11 | 6.14 | 19.34 | 9.42 | 17.37 | 6.99 |
| Effector CD4 T Cells | 1.62 | 3.53 | 1.42 | 2.13 | 2.06 | 3.57 | 0.98 | 1.09 | 0.26 | 0.43 | 1.41 | 1.57 | 2.20 | 3.27 | 1.05 | 1.30 | 1.26 | 0.96 | 1.86 | 3.14 |
| Regulatory T Cells | 1.05 | 0.48 | 0.83 | 0.58 | 1.23 | 0.63 | 0.81 | 0.39 | 1.98 | 1.78 | 0.78 | 0.46 | 1.05 | 0.37 | 0.97 | 0.64 | 0.97 | 0.72 | 1.38 | 0.62 |
| Non classical Monocytes | 0.54 | 0.62 | 0.12 | 0.17 | 0.36 | 0.38 | 0.70 | 0.88 | 0.40 | 0.38 | 0.08 | 0.09 | 0.82 | 0.45 | 0.13 | 0.12 | 0.40 | 0.31 | 0.52 | 0.41 |
| Intermediate Monocytes | 0.40 | 0.67 | 0.09 | 0.10 | 0.12 | 0.08 | 0.29 | 0.52 | 0.06 | 0.05 | 0.05 | 0.05 | 0.30 | 0.26 | 0.13 | 0.09 | 0.17 | 0.19 | 1.67 | 0.93 |
| Classical Monocytes | 18.53 | 9.95 | 18.78 | 14.49 | 9.13 | 5.42 | 11.76 | 7.08 | 10.31 | 0.95 | 14.03 | 8.20 | 13.73 | 7.52 | 17.78 | 14.61 | 11.37 | 6.44 | 12.04 | 5.26 |
| Dendritic Cells | 2.87 | 8.69 | 2.95 | 9.42 | 0.92 | 0.60 | 1.03 | 0.64 | 1.46 | 0.64 | 1.44 | 1.15 | 0.93 | 0.35 | 1.40 | 1.10 | 0.63 | 0.30 | 0.44 | 0.34 |
| Unidentified cells | 4.06 | 2.24 | 4.17 | 6.80 | 2.77 | 1.94 | 3.79 | 3.04 | 5.01 | 1.24 | 2.82 | 1.54 | 2.68 | 1.35 | 3.40 | 4.55 | 2.73 | 2.00 | 1.03 | 0.57 |

## Supplementary Table 5.

Forward selection linear regression analysis for tumor mass.

|  | **Model excluding dexamethasone** | | | | **Model including dexamethasone** | | | |
| --- | --- | --- | --- | --- | --- | --- | --- | --- |
|  |  | **95% Confidence interval** | |  |  | **95% Confidence interval** | |  |
| **Tumor mass (log10)** | **B** | **Lower bound** | **Upper bound** | **p-value** | **B** | **Lower bound** | **Upper bound** | **p-value** |
| NK Cells | -0.026 | -0.048 | -0.005 | 0.018 |  |  |  |  |
| Non-classical Monocytes | -0.306 | -0.566 | -0.046 | 0.022 |  |  |  |  |
| B memory cells |  |  |  |  | -1.111 | -1.659 | -0.130 | 0.045 |
| Age | -0.004 | -0.012 | 0.004 | 0.275 | -0.033 | -0.010 | 0.005 | 0.542 |
| Sex | -0.040 | -0.249 | 0.169 | 0.702 | -0.002 | -0.225 | 0.160 | 0.736 |
| Dexamethasone use before surgery |  |  |  |  | 0.524 | 0.309 | 0.74 | 0.000 |

# Supplementary figures


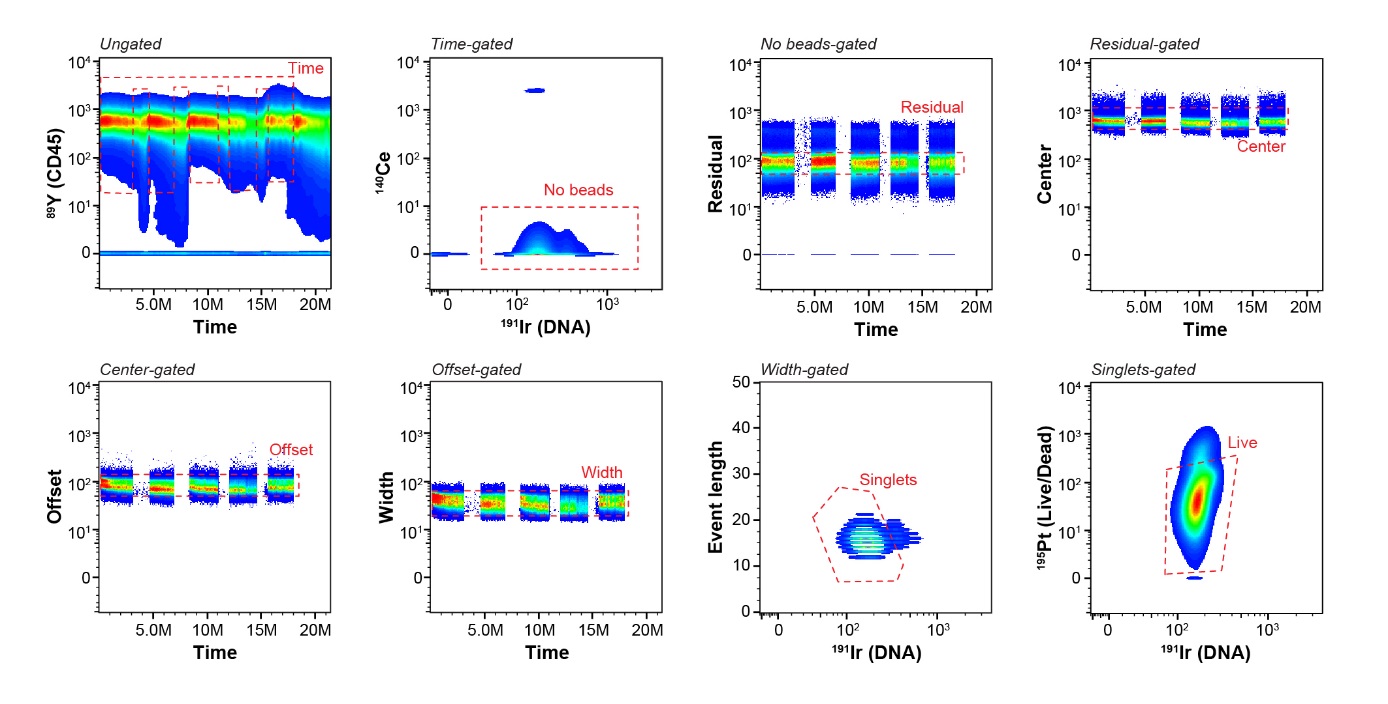


Supplementary figure 1. Gating of CD45+ live cells. Events were first gated based on stability of signal across time and CD45 positivity. Next, beads were excluded and the resulting events further gated according to their Gaussian parameters, as recommended by Bagwell et al. The remaining events were gated for single live events using 191Ir, 195Pt, and event length.


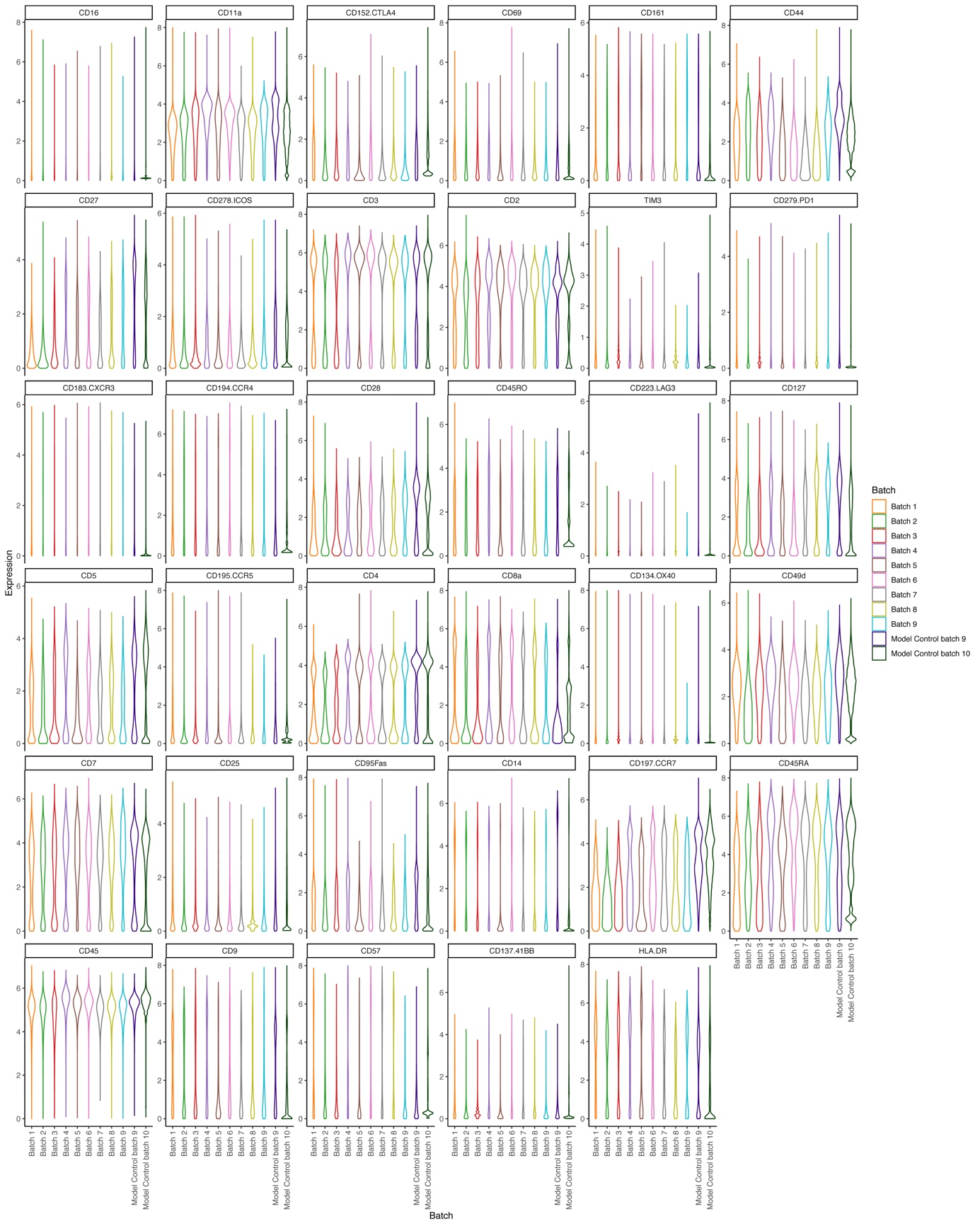
 Supplementary figure 2. Marker distribution before batch normalization. First 9 samples are biological replicates. Model control was a separate sample from batch 9, repeated in batch 10.


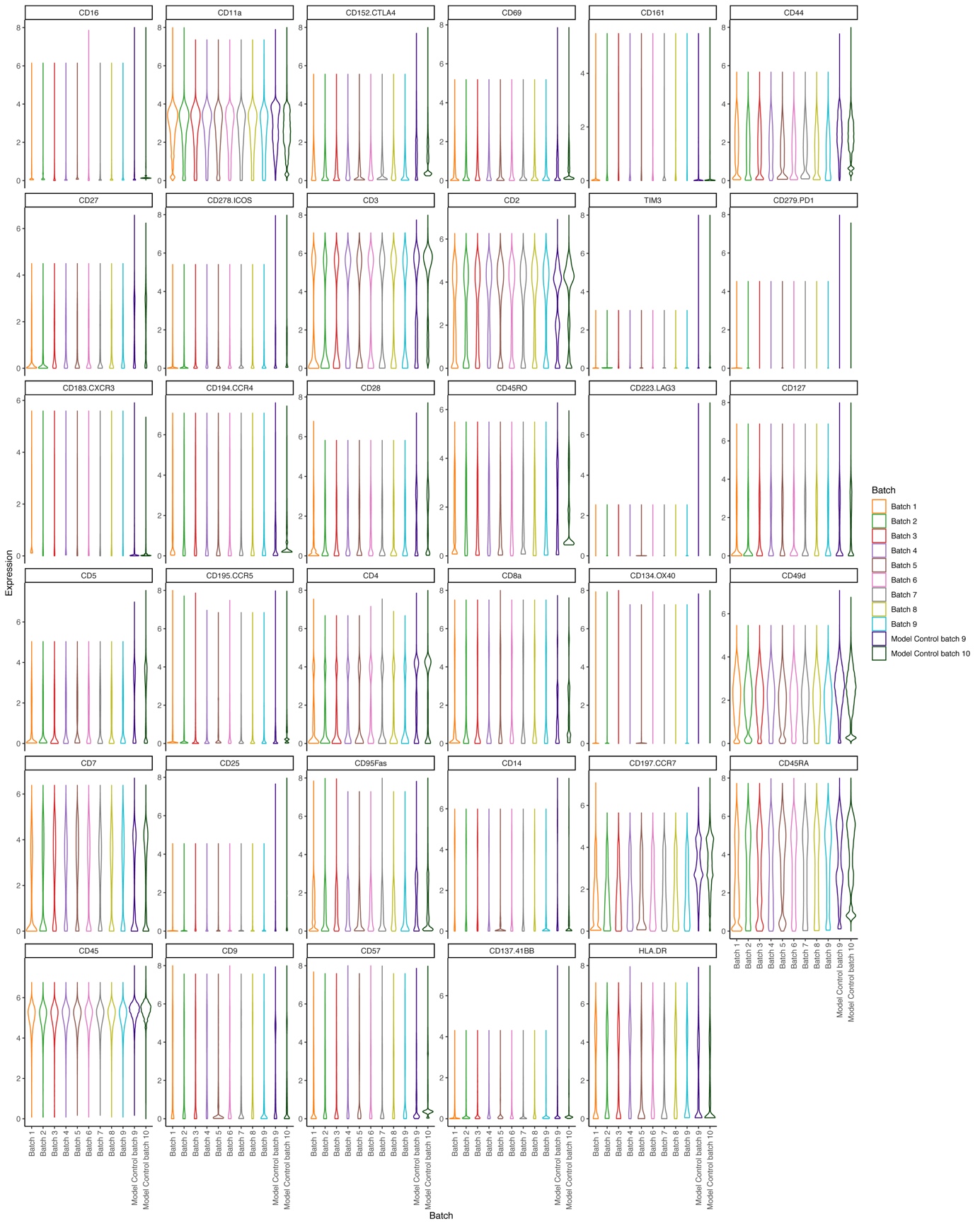
 Supplementary figure 3. Marker distribution after batch normalization. First 9 samples are biological replicates. Model control was a separate sample from batch 9, repeated in batch 10.


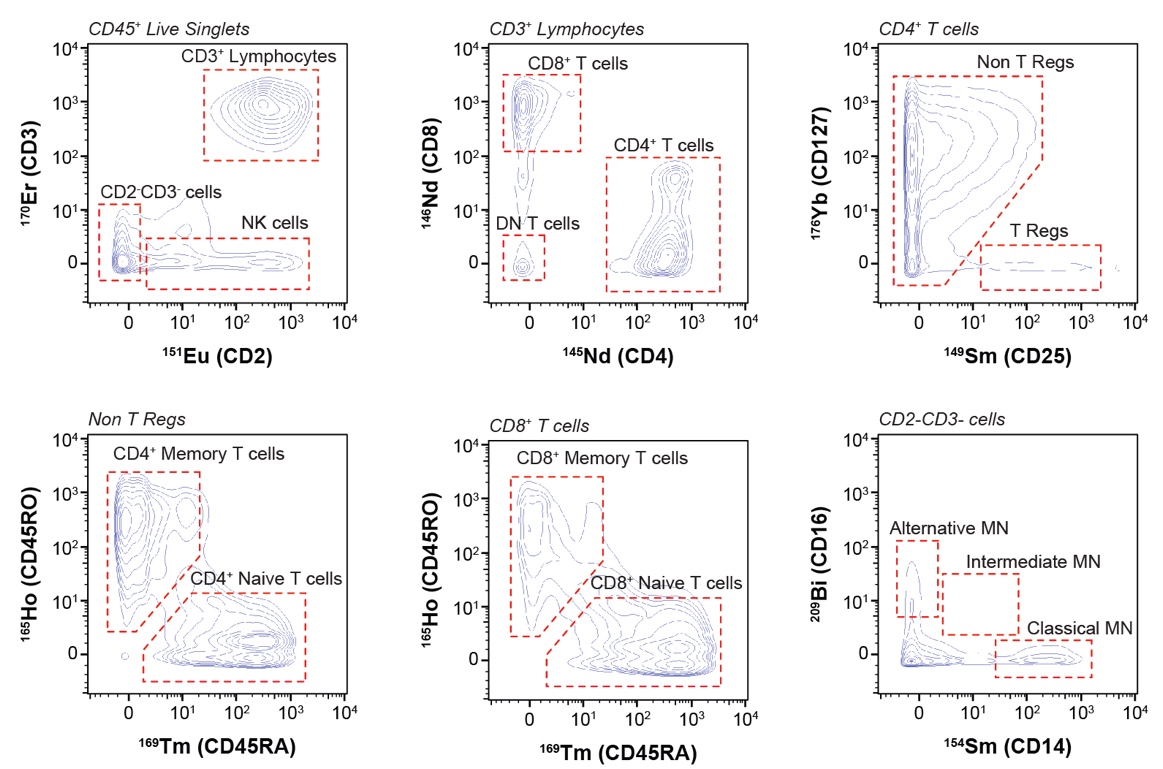


Supplementary figure 4. Almost all metaclusters identified in Fig. 2 can be manually gated with the help of CD2, CD3, CD4, CD8, CD127, CD25, CD45RA, CD45RO, CD14, and CD16.

**
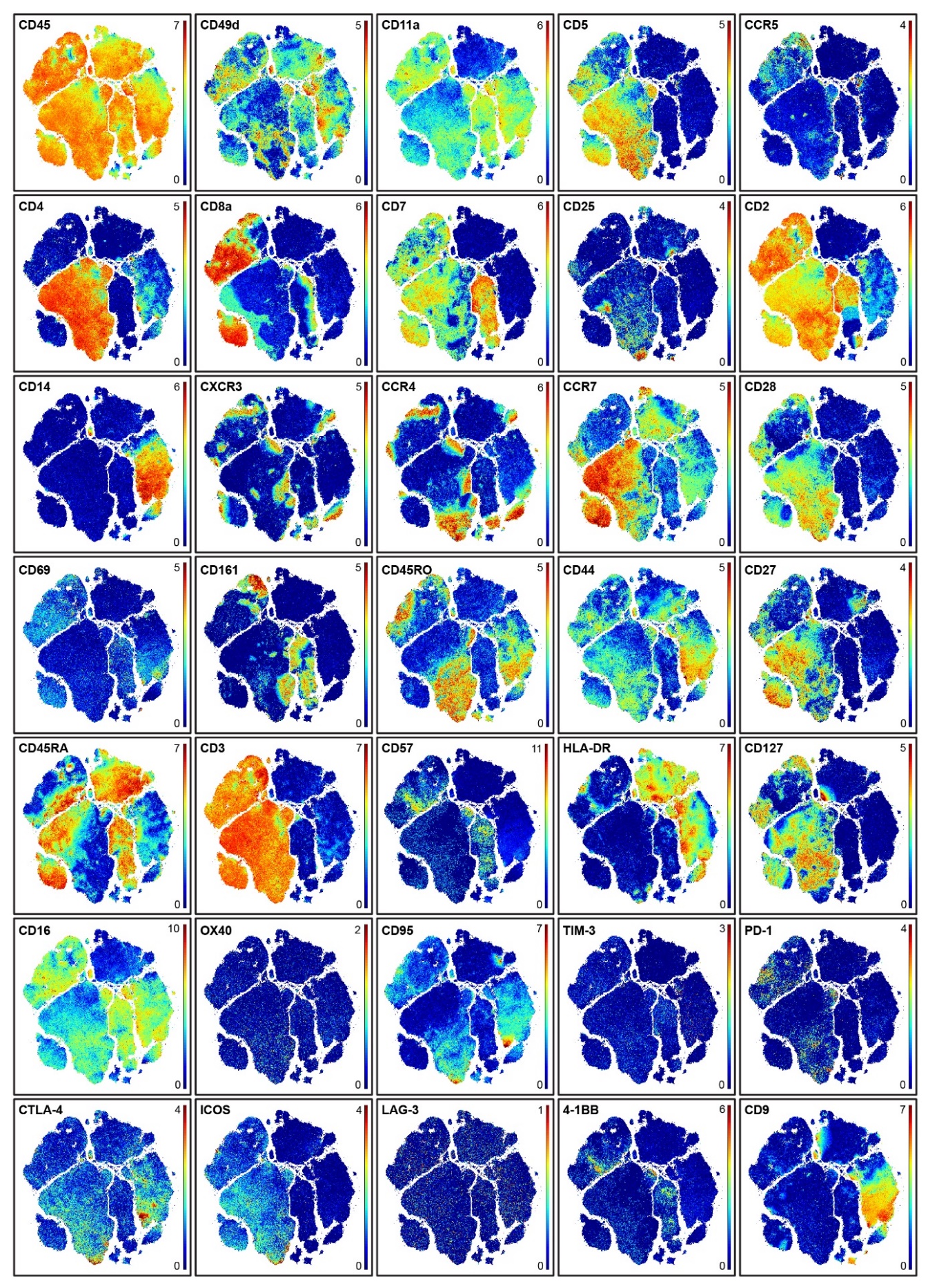
**

Supplementary figure 5. Coloured-continuous optSNE plots for each of the parameters determined.

**
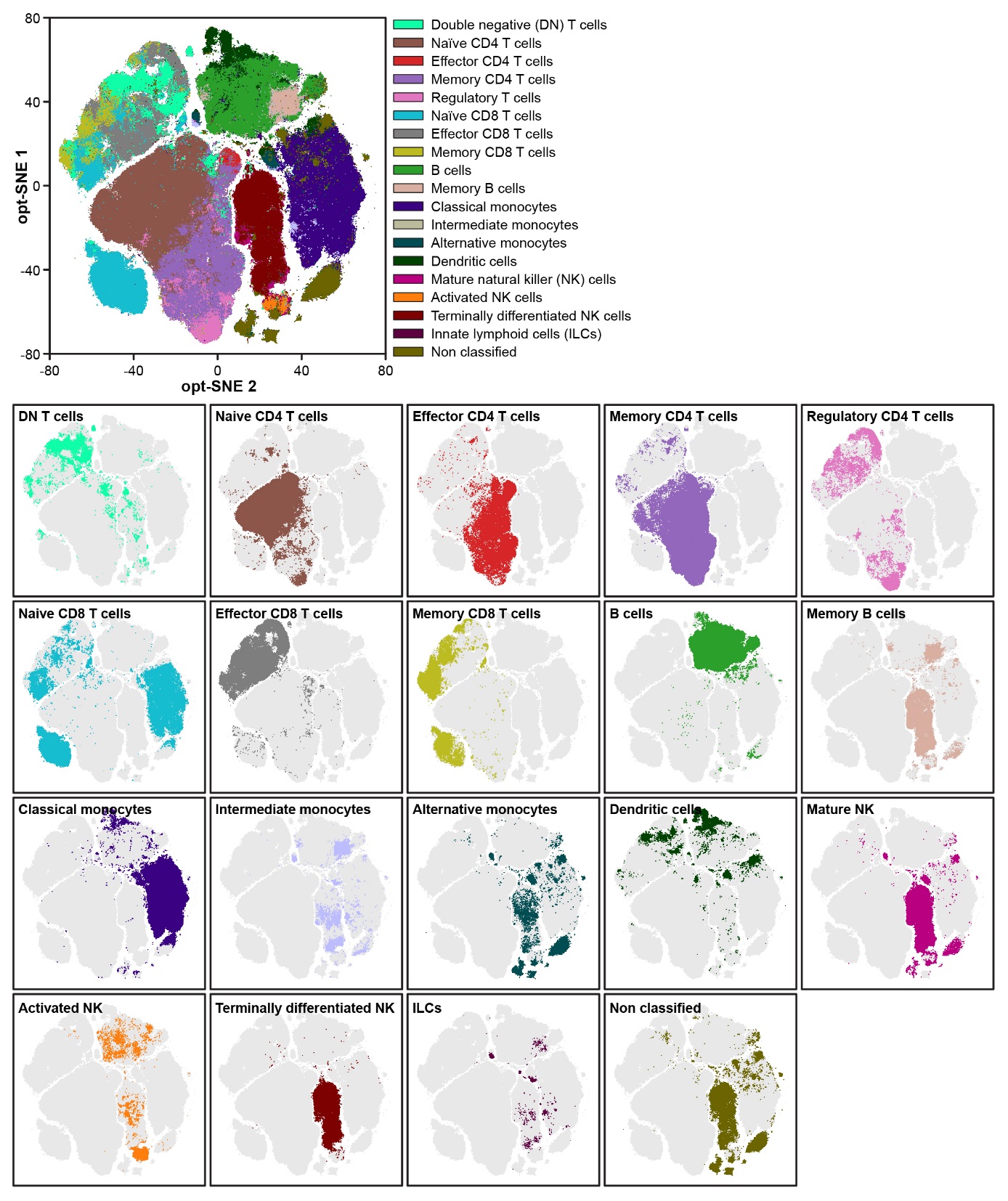
**

Supplementary figure 6. FlowSOM metacluster overlay on optSNE plots.

**
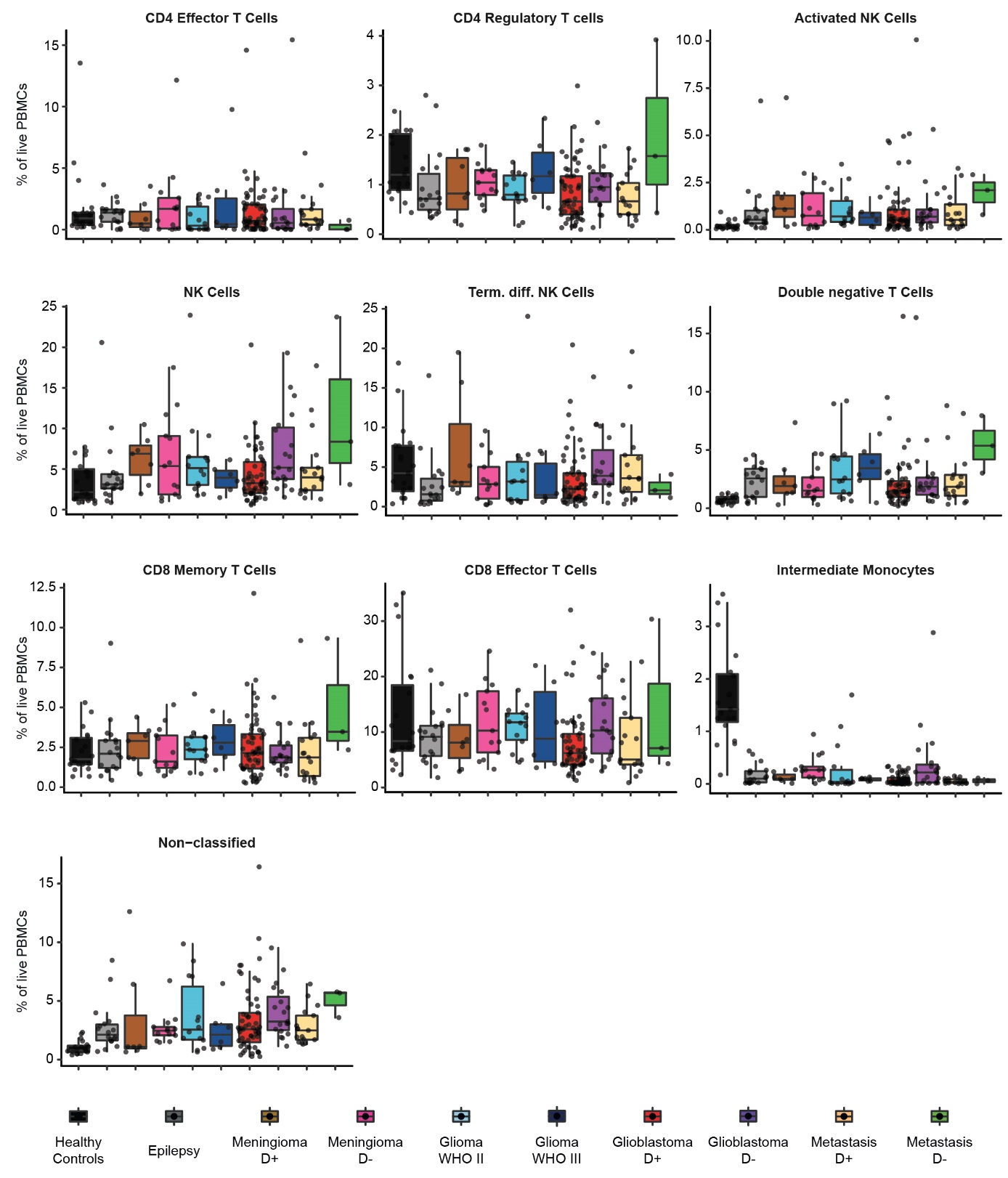
**

Supplementary figure 7. Frequencies of FlowSOM metaclusters not presented in Fig.5 for each patient group.

samples group based on dexamethasone treatment, more so than based on patient group.
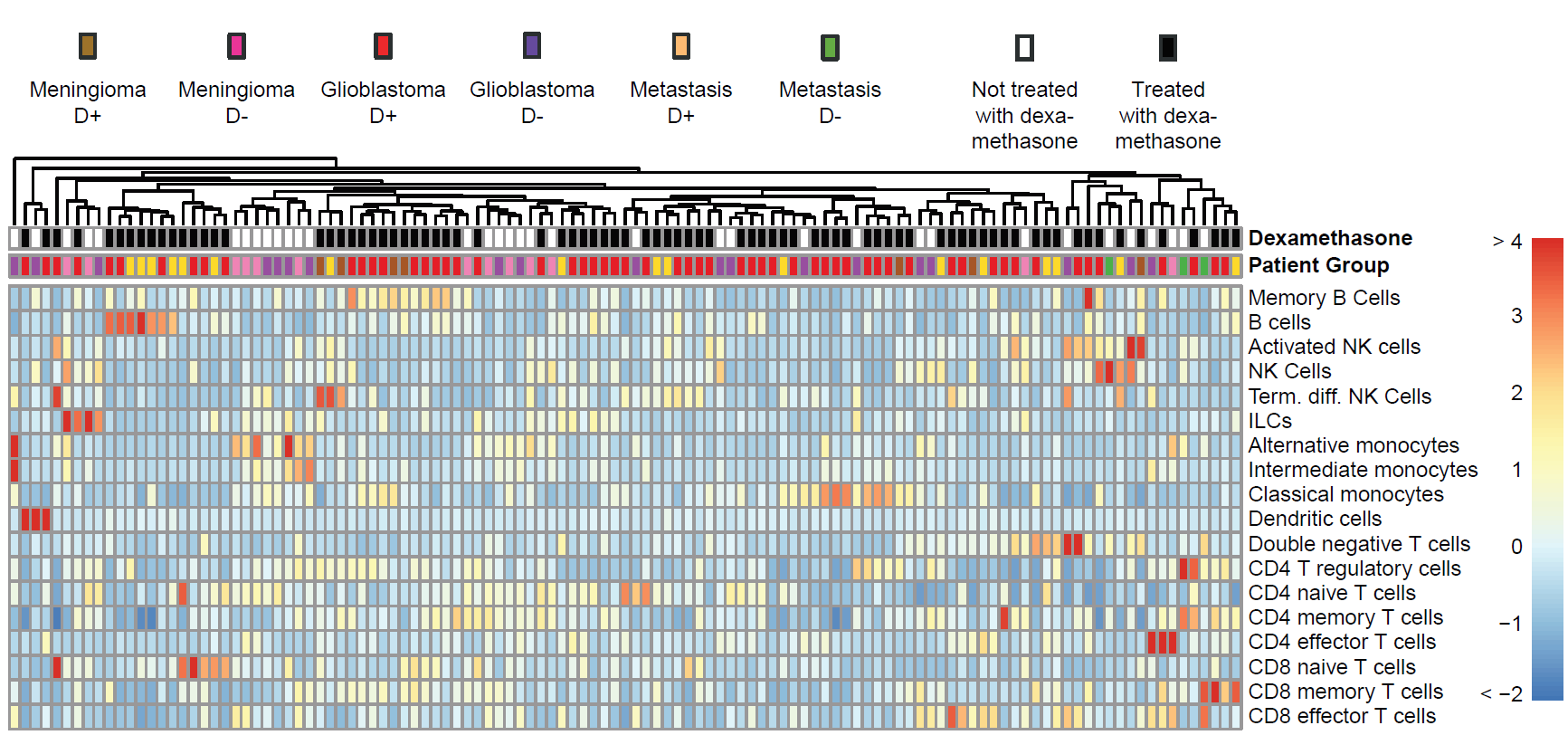
 Supplementary figure 8. Heatmap depicting cell type frequencies per patient in glioblastoma, meningioma and metastasis patient groups. Hierarchical clustering shows that samples group based on dexamethasone treatment, more so than based on patient group.

**
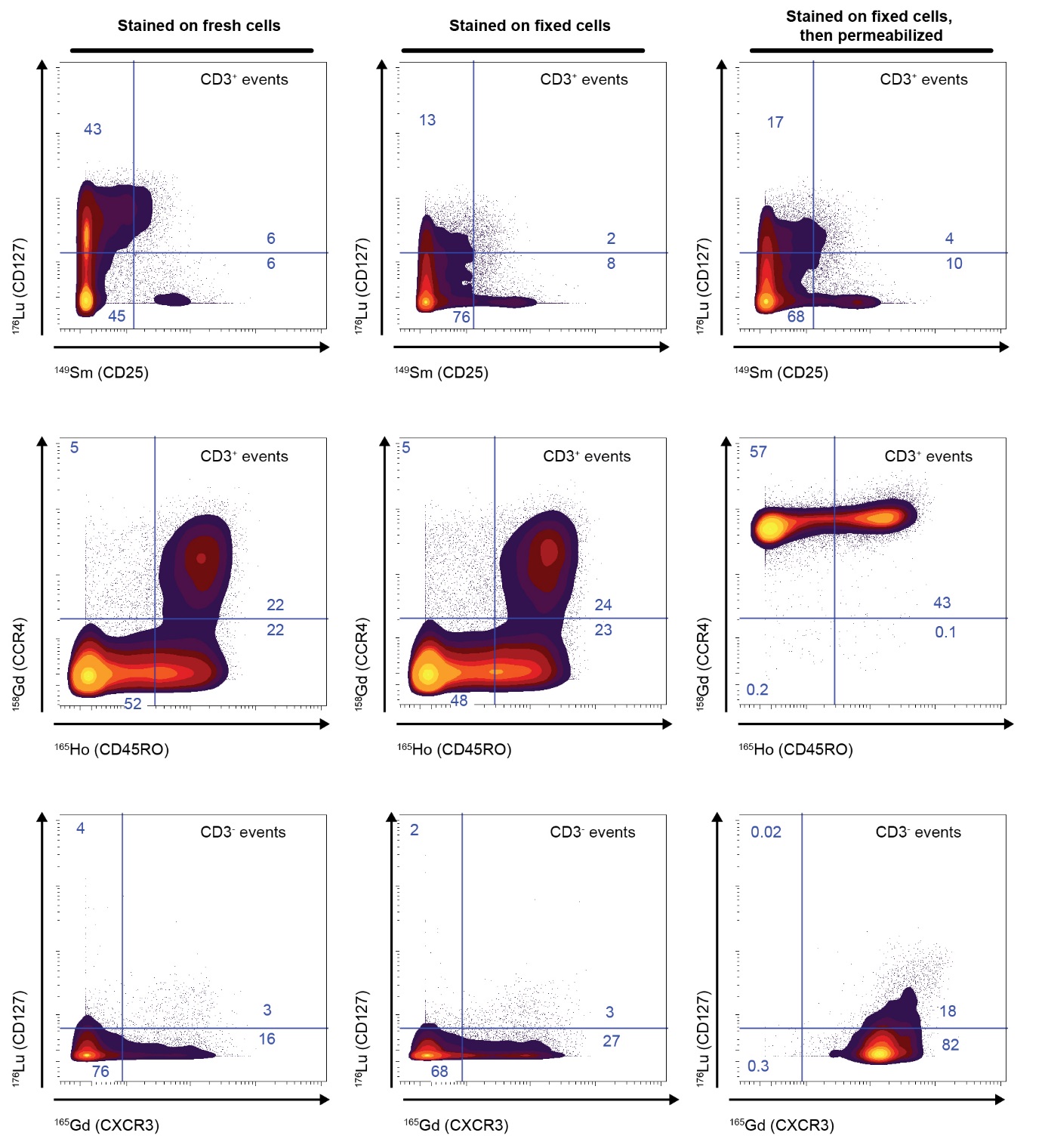
** Supplementary figure 9. Characterization of fixation-sensitive surface markers.
